# Selective expansion of myeloid and NK cells in humanized mice yields human-like vaccine responses

**DOI:** 10.1038/s41467-018-07478-2

**Published:** 2018-11-28

**Authors:** Florian Douam, Carly G. K. Ziegler, Gabriela Hrebikova, Bruno Fant, Robert Leach, Lance Parsons, Wei Wang, Jenna M. Gaska, Benjamin Y. Winer, Brigitte Heller, Alex K. Shalek, Alexander Ploss

**Affiliations:** 1 Department of Molecular Biology, Lewis Thomas Laboratory, Princeton University, Washington Road, Princeton, NJ 08544 USA; 2 Institute for Medical Engineering & Science (IMES), MIT, Cambridge, MA 02139 USA; 3 Department of Chemistry, Massachusetts Institute of Technology, Cambridge, MA 02142 USA; 4 Koch Institute for Integrative Cancer Research, Massachusetts Institute of Technology, Cambridge, MA 02139 USA; 5 Broad Institute of MIT and Harvard, Cambridge, MA 02142 USA; 6 Ragon Institute of MGH, MIT and Harvard, Cambridge, MA 02142 USA; 7 Harvard-MIT Division of Health Sciences and Technology, Harvard Medical School, Boston, MA 02139 USA; 8 Graduate Program in Biophysics, Harvard Medical School, Boston, MA 02139 USA; 9 Department of Psychiatry, Center for Neurobiology and Behavior, Perelman School of Medicine at the University of Pennsylvania, Philadelphia, PA 19104 USA; 10 Lewis Sigler Institute for Integrative Genomics, Genomics Core, Carl Icahn Laboratory, Princeton University, Princeton, NJ 19104 USA

## Abstract

Mice engrafted with components of a human immune system have become widely-used models for studying aspects of human immunity and disease. However, a defined methodology to objectively measure and compare the quality of the human immune response in different models is lacking. Here, by taking advantage of the highly immunogenic live-attenuated yellow fever virus vaccine YFV-17D, we provide an in-depth comparison of immune responses in human vaccinees, conventional humanized mice, and second generation humanized mice. We demonstrate that selective expansion of human myeloid and natural killer cells promotes transcriptomic responses akin to those of human vaccinees. These enhanced transcriptomic profiles correlate with the development of an antigen-specific cellular and humoral response to YFV-17D. Altogether, our approach provides a robust scoring of the quality of the human immune response in humanized mice and highlights a rational path towards developing better pre-clinical models for studying the human immune response and disease.

## Abstract (web summary)

Humanized mice are an enabling technology to explore human immunity and disease. Here, Douam et al. provide an in-depth comparison of immune responses to yellow fever vaccine in human vaccinees, conventional and second-generation humanized mice and define a workflow to evaluate and refine these models.

## Introduction

Much has been learned about how the mammalian immune system functions at steady state and during infection using inbred mouse models. However, it has become increasingly recognized that the mouse and human immune systems differ in numerous important aspects^1^, thus limiting the predictive value of studies in rodents for human biology. Furthermore, the narrow host tropism of many important human-tropic pathogens precludes the use of conventional mouse models for analyzing the interactions of such pathogens with the mammalian immune system^2^. The direct study of human immune responses is challenging as usually only peripheral blood, but not material from lymphoid organs or the site of infection, is readily accessible. Immune responses to many pathogens have been studied in patients, but interpreting such clinical data is difficult as numerous parameters that could influence measured immune response are often unknown. To gain better control of these critical factors, immune responses to live-attenuated vaccines, including yellow fever^3^, flu^4^, and smallpox^5^, have been carefully characterized. These studies have greatly contributed to our understanding of human immunity, but intra- and inter-donor variability, previous and/or current infections, age or microbiotic status still add significant complexity to the data and make analysis challenging.

Humanized mice have emerged as powerful tools for studying a broad range of human(-tropic) pathogens. Mice engrafted with components of a human hematolymphoid system or human immune system (HIS) have been especially useful for dissecting the interactions of human viruses with human immune cells^6–10^. A variety of mouse strains (reviewed in ref. ^11^) well-suited for engraftment of human hematolymphoid cells have been developed. These recipient strains are usually highly immunocompromised to facilitate engraftment of xenogeneic cells. Non-obese diabetic (NOD) mice deficient for both the recombinase activating gene 1 (Rag1^−/−^) and the IL-2 receptor gamma chain (IL2Rγ^null^) (NRG mice) are commonly used and do not develop functional murine B, T, or natural killer (NK) cells^12^. NRG mice are also deficient in hemolytic complement^13^ and harbor a polymorphism in the gene encoding murine signal regulatory protein α (SIRPα), which reduces phagocytic activity against human cells^14^. Injection of irradiation-conditioned NRG mice with human hematopoietic stem cells (HSCs) leads to de novo hematopoiesis, resulting in stable engraftment of human hematolymphoid system components^6,12,15^.

Although there is evidence that the engrafted HIS in such mice becomes activated upon microbial challenge, the quality of the immune response in conventional models and in other refined models (such as the bone marrow–liver–thymus, or BLT model) remains weak or uncertain^7,9,16–20^. One of the major reasons is the underrepresentation of critical human immune cell lineages in these models, which are crucial for activating the adaptive immune response. In particular, the scarcity of human dendritic cells (DCs) as well as other myeloid lineages and NK cells, decreases the functionality of the engrafted HIS. The small frequencies of these cell populations can be explained, in part, by the limited biological cross-reactivity of the non-redundant cytokines that promote lineage differentiation^21^. Consequently, several new humanized mice models with significant reconstitution of myeloid and/or NK cell compartments have been recently developed (hereon referred to as second-generation humanized mouse models). Indeed, exogenous administration of human interleukin (IL) 15 or an IL15/IL15 receptor (R) fusion protein significantly increases human NK cell numbers^22^. Similarly, injection of recombinant cytokines, such as granulocyte-macrophage colony stimulating factor (GMCSF), macrophage colony stimulating factor (MCSF), IL3 or FMS-like tyrosine kinase 3 ligand (Flt3LG), or their expression in engineered xenorecipient strains results in increased frequencies of erythro-myeloid lineage cells^15,23–25^. However, knowledge of how the human immune response in any of these novel models compares to those observed in humans remains limited.

To address this need, we devised an experimental pipeline allowing us to quantitatively assess immunity in humanized mice and compare to host responses in humans. By probing the cellular, humoral, and transcriptomic response to a highly immunogenic common standard, the yellow fever virus vaccine YFV-17D^26^, we provide here the first comprehensive comparison of the human immune response in conventional, second-generation humanized mice and human vaccinees. Our results highlight that selective expansion of myeloid and NK cells in humanized mice induces transcriptomic responses to YFV-17D infection akin to those of human vaccinees. The more human-like transcriptomic responses, lacking in conventional models, correlated with the development of antigen-specific cellular and humoral immunity to YFV-17D in humanized mice more robustly engrafted with human NK and various myeloid cells. Altogether, our work demonstrates a robust approach for the quantitative measurement of immunity in humanized mice, for more objective model cross-comparison and consequently, for the rational development of better pre-clinical humanized models.

## Results

### Conventional humanized mice mount limited immunity to YFV-17D

**Fig. 1:**
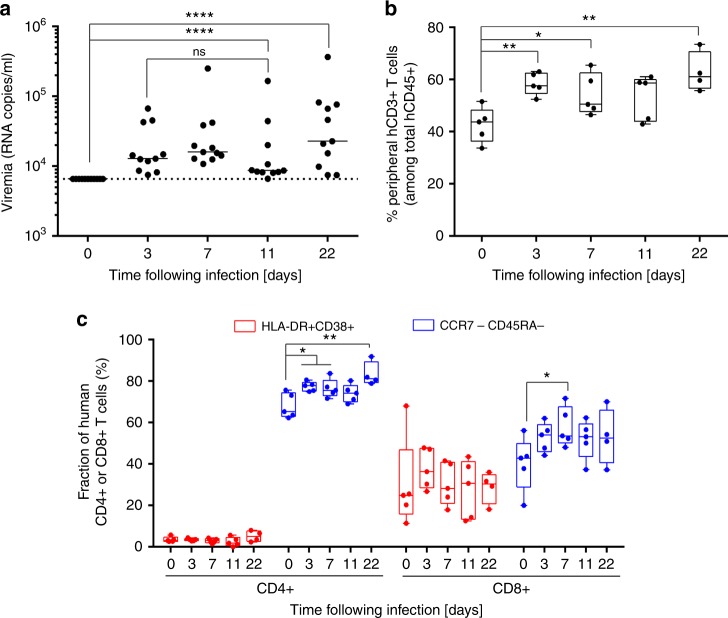
NRG-HIS mice do not clear YFV-17D infection. **a** YFV-17D serum viremia in the peripheral blood of NRG-HIS mice over the course of infection. (+) RNA copies per ml were quantified by RT-qPCR. Limit of detection (dotted line) is shown. Horizontal lines represent median viremia at each time point (*n* = 12). ****p ≤ 0.0001, ns non-significant (Wilcoxon–Mann–Whitney test). **b** Percentage of peripheral human CD3+ T cells among the total human CD45+ cell population in the blood of NRG-HIS mice over the course of YFV-17D infection. Bounds of box and whiskers represent the min-to-max fraction of peripheral human CD3+ T cell among total human CD45+ at each time point. Medians are indicated in each box as center line (*n* = 5) *p ≤ 0.05, **p ≤ 0.01 (Student’s *t* test). **c** Fraction of peripheral human CD4+ and CD8+ T cells co-expressing HLA-DR and CD38 (red) or lacking expression of both CCR7 and CD45RA (blue) in the blood of NRG-HIS mice over the course of YFV-17D infection. Bounds of box and whiskers represent the min-to-max fraction of human CD4+ or CD8+ T cell for each marker combination and time point. Medians are indicated in each box as center line (*n* = 5). *p ≤ 0.05, **p ≤ 0.01 (Student’s *t* test)

YFV-17D is one of the most potent vaccines ever developed, and single vaccination usually results in protection for at least 10 years^26^. Existing data on the immune response to YFV-17D in human vaccinees could thus serve as a valuable comparator to systematically assess the functionality of a transplanted HIS. In contrast to the transient or even undetectable viremia observed in human vaccinees^3,5^, YFV-17D RNA rapidly reached a plateau and persisted in the blood of NRG-HIS mice for at least 22 days, suggesting the engrafted HIS cannot effectively clear infection (Fig. 1a) despite an absence of significant mortality (Supplementary Figure 1a). In YFV-17D-infected conventional NRG-HIS mice, we noticed an overall increase in peripheral CD3+ T cells upon YFV-17D infection (Fig. 1b) without any changes in the ratio of CD4+/CD8+ T cells (Supplementary Figure 1b). In contrast to reports in patients, the frequencies of human CD8+ T cells expressing HLA-DR and CD38—two markers to track virus-activated cells within the bulk CD8+ T cell population in the peripheral blood of human vaccinees^5^—did not change in the blood (Fig. 1c) or spleen (Supplementary Figure 1c) of these mice. Downregulation of CCR7 and CD45RA on a subset of CD4+ and CD8+ T cells was detectable in the blood and spleen of NRG-HIS mice upon YFV-17D infection (Fig. 1c; Supplementary Figure 1c), indicating that the engrafted HIS responded to the infection. However, this activation did not correlate with better control of viral replication in the periphery (Fig. 1a). To enable tracking of antigen-specific T cells responses, we infected humanized NRG mice expressing transgenically HLA-A2*0201 (NRG-A2-HIS) and quantified YFV-specific CD8+ T cells in the blood and spleen of NRG-A2-HIS mice using an HLA-A2:YFV NS4B (amino acids 214–222, LLWNGPMAV) tetramer^3^. Unlike previous studies characterizing virus-specific CD8+ T cell responses to HIV, Epstein-Barr virus, dengue virus, or adenovirus^9,17,19,27^, we did not detect any A2:LLWNGPMAV-specific T cells in either the blood or spleen over the course of infection, indicating that YFV-17D-specific cells are poorly primed in NRG-A2-HIS mice (Supplementary Figure 1d). These data indicate that while NRG-HIS mice have utility as a challenge model^6^, they require further refinements to better model human immune responses.

**Fig. 2:**
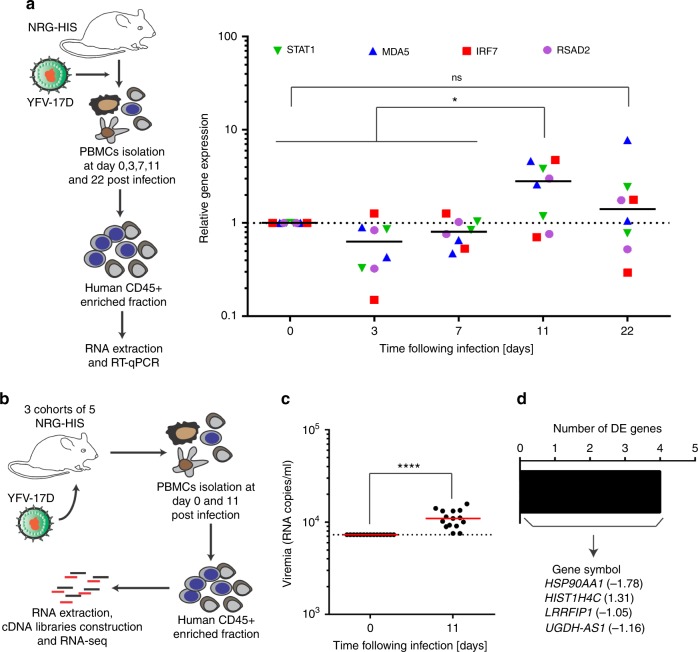
Limited transcriptomic response to YFV-17D infection in NRG-HIS mice. **a** Relative expression of a set of four anti-viral genes (green, *STAT1*; blue, *MDA5*; red, *IRF7*; and purple, *RSAD2*) in the PBMCs of NRG-HIS mice following infection with YFV-17D. Expression of each gene was assessed by RT-qPCR in human peripheral CD45+ cells at different time points post infection (day 0, 3, 7, 11, and 22 post infection). Each dot represents the average expression of a given gene within a cohort of 4 NRG-HIS mice. For each time point, the grand median is shown and represent the median of the cumulated expression of the four genes. Dotted line represents the gene expression level at baseline (*n* = 2 cohorts of four NRG-HIS mice each). *p ≤ 0.05, ns non-significant (two-way ANOVA). **b** Schematic representation of the procedure to characterize the PBMC transcriptomic signature of NRG-HIS mice following YFV-17D infection. **c** YFV-17D serum viremia at days 0 and 11 post infection in the NRG-HIS mice used for transcriptomic profiling. (+) RNA copies per ml were quantified by RT-qPCR. Red horizontal lines represent median viremia at each time point. Limit of detection (dotted line) is shown (*n* = 15). ****p *≤* 0.001 (Wilcoxon–Mann–Whitney test). **d** Number of significantly differentially expressed (DE) genes (*p*_adj_ ≤ 0.05) in the PBMCs of NRG-HIS mice following YFV-17D infection. The names of the only four DE genes is depicted, followed with their respective log_2_ fold change

Next, we examined the expression of four genes (*MDA5/IFIH1*, *STAT1*, *IRF7*, *RSAD2*), all reported to be upregulated in PBMCs of human vaccinees following vaccination^28^, in the human PBMCs of NRG-HIS mice following YFV-17D infection. Since we expected the anti-viral response in NRG-HIS to be low, we aimed at determining the cumulative median expression of these four anti-viral genes by RT-qPCR using human-specific primers. Our data revealed that the cumulative median expression of these four genes significantly increased at day 11 post infection relative to pre-infection levels (day 0) (Fig. 2a; Supplementary Figure 1e). Using RNA-sequencing (RNA-seq), we then performed an unbiased quantification of differentially expressed genes in human PBMCs of NRG-HIS mice on day 11 post YFV-17D infection. Notably, only four genes were significantly differentially expressed (DE) (*HSP90AA1*, *HIST1H4C*, *LRRFIP1*, and *UGDH-AS1*) (Fig. 2b–d; Supplementary Data 1), highlighting the limited and variable transcriptomic response of human PBMCs in NRG-HIS mice. Altogether, our results indicate that NRG-HIS develop an extremely limited human immune response to YFV-17D.

### Enhanced human myeloid and NK cell reconstitution in HIS mice

**Fig. 3:**
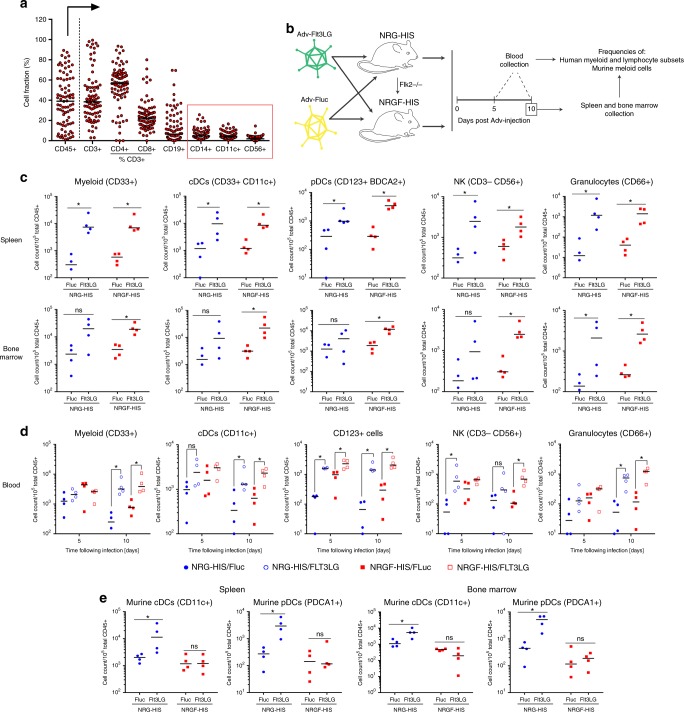
Selective expansion of human myeloid cells and NK cells in humanized mice. **a** Immune system reconstitution in NRG-HIS mice. Frequency of each cell fraction is shown as a percentage of CD45+ cells, with the exception of CD4+ and CD8+ T cells, which are displayed as a percentage of CD3+ T cells. The frequencies of important myeloid subsets (CD14+ monocytes and CD11c+ dendritic cells) and CD56+ NK cells are highlighted by a red box. Medians are shown for each cell subset frequency as horizontal black line (*n* = 82). **b** Schematic representation of the experimental procedure employed to evaluate the ability of NRGF-HIS mice to selectively expand human myeloid cell subsets. 10^11^ AdV-Fluc or 10^10^ AdV-Flt3LG particles were injected into NRG-HIS or NRGF-HIS mice, and immune cell expansion was examined at day 5 and 10 post Adv-injection. **c**, **d** Expansion of human immune cell subsets in the spleen (**c**), bone marrow (**c**) or blood (**d**) of NRG-HIS and NRGF-HIS mice. NRG-HIS (blue circle) and NRGF-HIS (red square) mice were injected with AdV-Fluc (closed circle/square in panel **c**) or AdV-Flt3LG (open circle/square in panel **c**). **e** Expansion of murine cDCs and pDCs in the spleen and bone marrow of NRG-HIS and NRGF-HIS mice following injection with AdV-Flt3LG or AdV-Fluc. For panels (**c**–**e**), medians are shown as horizontal black lines for each experimental condition (*n* = 4 per group). *p ≤ 0.05, ns non-significant (Wilcoxon–Mann–Whitney test). cDCs conventional dendritic cells, pDCs plasmacytoid dendritic cells, NK natural killer cells

Impaired immune function in conventional NRG-mice (reviewed in ref.^29^) can likely be attributed in part to low frequencies of critical immune cell subsets, such as myeloid and NK cells, compared to humans (Fig. 3a) where the myeloid compartment represents 50–80% of peripheral leukocytes. Since DCs and NK cells are key effectors of the innate immune response and critical in the activation of an adaptive response, we hypothesized that selective expansion of these cell subsets in humanized mice could promote an enhanced human immune response.

Receptor-type tyrosine-protein kinase FLT3, or fetal liver kinase-2 (Flk2), is a cell surface receptor broadly expressed on early hematopoietic precursors in the bone marrow. Consequently, myeloid cell development is severely impaired in Flk2-deficient mice (*Flk2*^−/−^)^30,31^. However, in xenorecipient mouse strains commonly used for human hematopoietic engraftment^12,32–34^, murine myelopoiesis is largely unaffected. Thus, the majority of myeloid cells are still of murine origin and putatively interfere with priming of human-specific adaptive immune responses upon infection with (human-tropic) pathogens.

Despite the recent generation of humanized mouse models harboring a Flk2 deletion^25,35^, the influence of this on the human immune response in humanized mice has not yet been described nor directly compared to immunity in humans. Therefore, as a proof-of-concept, we aimed to quantitatively evaluate the impact of this enhancement on the cellular and transcriptomic response to YFV-17D infection through cross-comparison with other humanized mouse models and human clinical data.

We thus generated Flk2^−/−^ mice on the NRG background, yielding NRGF mice. While previous work relied on repeated injections of recombinant Flt3LG^24,25^, we chose a vectored delivery approach, constructing a replication-incompetent adenoviral system for sustained and stable production of human Flt3LG (AdV-Flt3LG) in HEK293T cells in vitro (Supplementary Figure 2a) and NRG mice in vivo (Supplementary Figure 2b). Following injection of NRG-HIS and NRGF-HIS mice with AdV-Flt3LG (NRG-HIS/Flt3LG or NRGF-Flt3LG, respectively) or with AdV-Fluc (NRG-HIS/Fluc or NRGF-HIS/Fluc, respectively) (Fig. 3b, Supplementary Figure 2c, d), we observed a significant expansion of CD33+ myeloid cells, CD33+ CD11c+ (conventional) DCs (cDCs), and CD123+ BDCA2+ plasmacytoid DCs (pDCs) in the spleen and/or bone marrow of NRG-HIS/Flt3LG mice (Fig. 3c, Supplementary Figure 2e, f; Supplementary Figure 3). Likewise, frequencies of CD56+ CD3− NK cells and CD66+ granulocytes increased in the spleen and bone marrow of NRGF-HIS/Flt3LG mice (Fig. 3c; Supplementary Figure 3). Moreover, several cellular lineages commonly under-represented in conventional humanized mice, including cDCs, monocytes, macrophages, granulocytes, NK cells and CD3+ CD56+ T cells (which include NK T cells and γδ T cells), were expanded in the peripheral blood (Fig. 3d; Supplementary Figure 2g; Supplementary Figure 3). Some of these human lineages were also more prevalent in NRG-HIS/Flt3LG mice than in NRG-HIS/Fluc mice but with greater variability across cohorts (Fig. 3c, d; Supplementary Figure 2f, g; Supplementary Figure 3). Importantly, the frequencies of various murine myeloid subsets, including cDC, pDC, and monocytes, remained largely constant in the spleen and bone marrow of NRGF-HIS/Flt3LG mice but were increased in NRG-HIS/Fltl3LG mice (Fig. 3e; Supplementary Figure 2h; Supplementary Figure 3), consistent with the fact that human Flt3LG is biologically cross-reactive and stimulates murine myeloid precursor cells^36^.

### Transcriptomic signatures to YF-17D in NRGF-HIS/Flt3LG mice

**Fig. 4:**
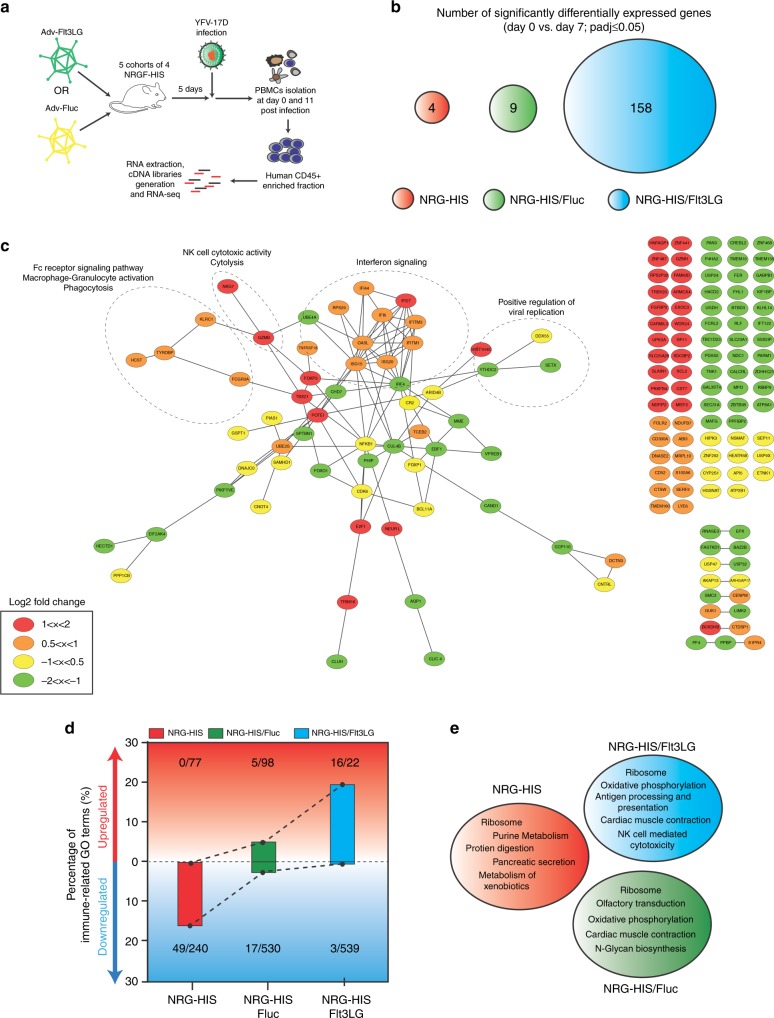
NRGF-HIS/Flt3LG mice display an extensive transcriptomic signature. **a** Schematic representation of the experimental procedure to characterize the PBMC transcriptomic signature of NRGF-HIS mice following YFV-17D infection. **b** Number of significantly DE genes (*p*_adj_ ≤ 0.05) at day 11 post YFV-17D infection (versus day 0, prior infection) in the PBMCs of NRG-HIS (red), NRGF-HIS/Fluc (green), and NRGF-HIS/Flt3LG (blue) mouse PBMCs upon YFV-17D infection. **c** Protein–protein network of significantly DE (*p*_adj_ ≤ 0.05) in NRGF-HIS mice following YFV-17D infection. Each gene is colored based on its log_2_FC (1 < *x* < 2, red; 0.5 < *x* < 1, orange; −1 < *x* < −0.5, yellow; −2 < *x* < −1, green). Areas enriched with genes related to a specific biological process are highlighted by a dotted circle or ellipse. **d** Frequencies of upregulated (red area) or downregulated (blue area) immune-related GO terms among all statistically significant GO terms (*p* ≤ 0.05) in NRG-HIS (red), NRGF-HIS/Fluc (green), and NRGF-HIS/Flt3LG (blue) mice following replicate analysis. Total count of immune-related GO-terms out of all significant GO terms (displayed as immune-related/all) are also reported. Dotted lines between the bars symbolize the progressive enhancement of human immune functionality across our humanized mice models. **e** KEGG pathway enrichment analysis of the transcriptomes of NRG-HIS (red), NRGF-HIS/Fluc (green), and NRGF-HIS/Flt3LG (blue) mouse PBMCs following replicate analysis. For each experimental setting or mouse model, the top five upregulated KEGG pathways are listed (*q* value ≤ 0.06)

By RNA-seq, NRGF-HIS/Flt3LG mice exhibited a signature of 158 genes significantly DE (*p* ≤ 0.05) while NRG-HIS and NRGF-HIS/Fluc mice displayed, respectively, 4 and 9 DE genes (Figs. 2d and 4a, b; Supplementary Figure 4a, b; Supplementary Data 1). The 158 genes in the NRGF-HIS/Flt3LG mice contained a panel of 65 upregulated genes among which were many immune response-related genes involved in macrophage activation/phagocytosis, NK cell cytotoxicity, or type I interferon (IFN) anti-viral response (Fig. 4c; Supplementary Fig. 4a). A gene ontology (GO) term enrichment analysis^37,38^ of the transcriptomic profiles of our different mouse models (following replicate analysis) showed that the total set of upregulated GO terms of NRGF-HIS/Flt3LG mice was enriched for immune-related GO terms (17% of total upregulated GO terms) in contrast to NRG-HIS (0%) and NRGF-HIS/Fluc mice (5%) (Fig. 4d; Supplementary Figure 5). In NRGF-HIS/Flt3LG mice, we identified upregulated GO terms related to IFN signaling, antigen presentation, and cytokine production, consistent with previous findings in humans^28,39^. Moreover, we observed an enrichment in GO terms related to the regulation of B and T cell-mediated immunity, providing additional evidence for a more comprehensive immune response to YFV17D in NRGF-HIS/Flt3LG mice. In contrast, 20% of the immune-related GO terms were significantly downregulated in NRG-HIS mice, further underscoring the limited functionality of the HIS in this model. A KEGG pathway^40,41^ enrichment analysis also confirmed the ability of NRGF-HIS/Flt3LG mice to mount an enhanced transcriptional response. Among the top five upregulated KEGG pathways identified in each mouse model, immune-related pathways were only found in NRGF-HIS/Flt3LG mice (Fig. 4e). These pathways, related to antigen presentation and NK cell activity, were consistent with our findings as well as with previous ex vivo and in vivo human studies^28,39,42,43^.

### Human-like transcriptomic responses in NRGF-HIS/Flt3LG mice

**Fig. 5:**
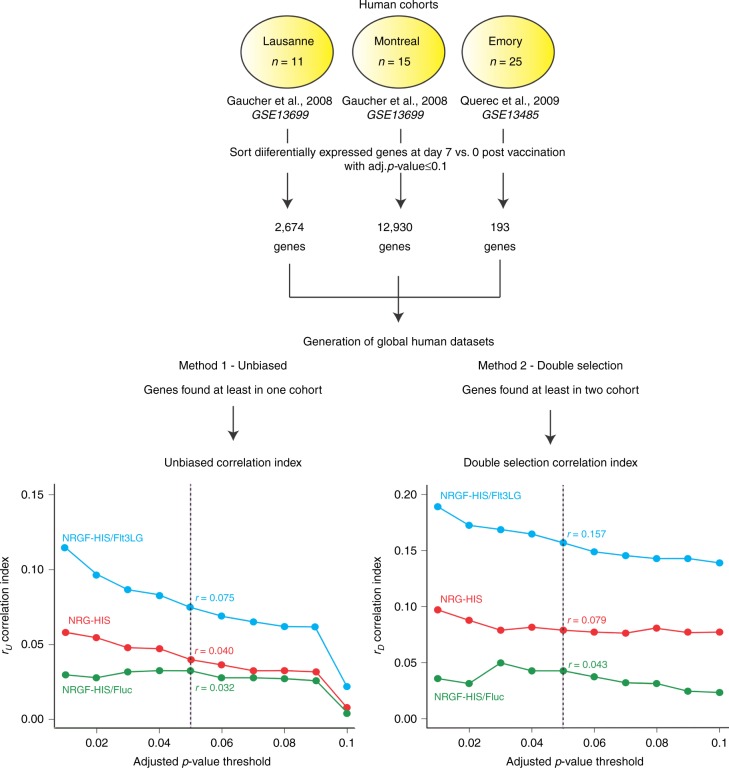
Human-like transcriptomic response to YFV-17D infection in NRGF-HIS/Flt3LG. Differentially expressed genes from PBMCs of three YFV-17D human vaccinee cohorts (Lausanne, *n* = 11; Montreal, *n* = 15; Emory, *n* = 25) were used to generate two global human transcriptomic datasets: an unbiased dataset (all differentially expressed genes (*p*_adj_ ≤ 0.1) found in at least one cohort) and an double-selection dataset (all differentially expressed genes (*p*_adj_ ≤ 0.1) found in at least two cohorts). For a given human global dataset (unbiased, left; double selection, right) and *p*_adj_ threshold (starting at *p*_adj_ ≤ 0.1 with 0.01 increment), the Spearman rho correlation index was computed for each humanized mouse model transcriptomic dataset (NRG-HIS, NRGF-HIS/Fluc, and NRGF-HIS/Flt3LG) and plotted as function of the *p*_adj_ threshold. The Spearman rho correlation index for the unbiased and double selection method are respectively referred as *r*_*U*_ and *r*_*D*_. The definitive correlation indexes for the unbiased and double selection method, referred as *r*_*U*_ and *r*_*D*_ at *p*_adj_ = 0.05 (*r*_*U,q*=0.05_ and *r*_*D,q*=0.05_) respectively, are indicated on each graph and for each humanized mouse model. See Methods for more details

Two studies have previously delineated the transcriptomic response to YFV-17D in human vaccinees^28,44^. Specifically, these studies identified sets of genes differentially regulated in PBMCs upon YFV vaccination. Hence, we utilized these valuable datasets as a reference to quantitatively evaluate how similar transcriptomic responses were in our different humanized mouse models in comparison to humans. We re-analyzed three datasets (hereon referred to as the Lausanne, Montreal, and Emory cohorts) derived from two independent studies^28,44^ and sorted the list of differentially regulated genes (*p*_adj_ ≤ 0.1) at day 7 post vaccination for each human cohort. From these three lists of genes, we then generated a global human dataset of genes differentially expressed upon YFV-17D vaccination using two distinct methods: an unbiased method and a double selection method, i.e., composed of genes found in at least one cohort, or in at least two cohorts respectively. Using these two distinct human reference datasets, we computed a Spearman rho correlation index (see Methods for details) for each humanized mouse model (NRG-HIS, NRGF-HIS/Fluc, and NRGF-HIS/Flt3LG; day 11 post vaccination) relative to human vaccinees, at varying *p*_adj_ thresholds (from 0.01 to 0.1 with 0.01 increments) for differentially expressed genes. Correlation indexes were referred to as *r*_*U*_ and *r*_*D*_ for the unbiased and double selection method respectively (see Methods for details), for a given *p*_adj_ threshold. We chose to use the correlation index value derived from gene sets constructed with an *p*_adj_ threshold of 0.05 as our definitive *r*_*U*_ and *r*_*D*_ correlation indexes (referred to as *r*_*U,q*__=0.05_ and *r*_*D,q*__=0.05_; see Methods for details). Independent of the method or *p*_adj_ threshold used, NRGF-HIS/Flt3LG always displayed the highest correlation index in comparison to the two other models (NRGF-HIS/Flt3LG *r*_*U,q*=0.05_ = 0.075 and *r*_*D,q*=0.05_ = 0.157 Fig. 5; Supplementary Table 1). When limiting the analysis to differentially expressed genes at *p*_adj_ ≤ 0.01, the *r*_*U*_ and *r*_*D*_ correlation indexes of the three mouse cohorts displayed the highest differences, with notably *r*_*D*_ for NRGF-HIS/Flt3LG reaching up to 0.188 (versus 0.0967 and 0.036 for NRG-HIS and NRGF-HIS/Fluc, respectively). Altogether, this analysis demonstrates the superiority of our NRGF-HIS/Flt3LG mice over conventional humanized mice for modeling the human transcriptomic response to YFV-17D infection. Moreover, our approach provides a valuable and relevant methodology for an accurate and objective scoring of human immunity in humanized mouse models.

### Increased control of viral infection in NRGF-HIS/Flt3L mice

**Fig. 6:**
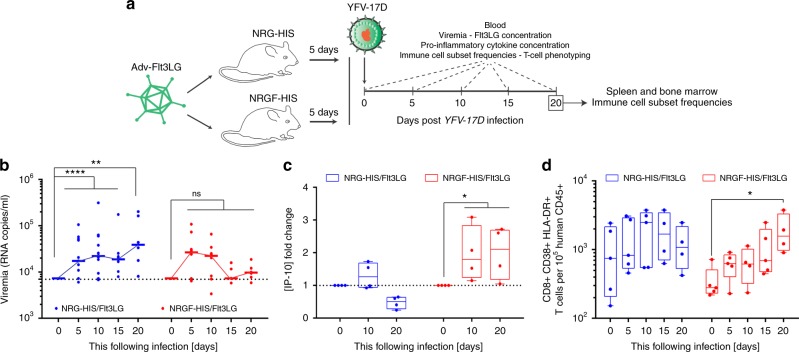
Improved control of infection and T-cell activation in NRGF-HIS/Flt3LG mice. **a** Schematic representation of the NRG-HIS and NRGF-HIS mice time course infection experiment. **b** YFV-17D serum viremia in the peripheral blood of NRG-HIS (blue, *n* = 12) and NRGF-HIS (red, *n* = 8) mice over the course of infection. (+) RNA copies per ml were quantified by RT-qPCR. Limit of detection (dotted line) is shown. Horizontal lines represent median viremia at each time point. **p ≤ 0.01, ****p ≤ 0.0001, ns non-significant (Wilcoxon–Mann–Whitney test). **c** IP-10 concentration fold-change in the serum of NRG-HIS (blue) and NRGF-HIS (red) mice over the course of infection. Bounds of box and whiskers represent the min-to-max concentration of IP-10 at each time point. Medians are indicated in each box as center line (*n* = 4–5 per group). *p ≤ 0.05, ***p ≤ 0.001, ns non-significant (Wilcoxon–Mann–Whitney test). **d** Frequencies of human CD3+ CD8+ HLA-DR+ CD38+ T cells in the blood of NRG-HIS (blue) and NRGF-HIS (red) mice over the course of YFV-17D infection. Bounds of box and whiskers represent the min-to-max frequencies of CD8+ HLA-DR+ CD38+ T cell at each time point. Medians are indicated in each box as center line (*n* = 5 per group). *p ≤ 0.05 (two-way ANOVA)

Next, we assessed how increased correlation index between human vaccinees and NRGF-HIS/Flt3LG mice related to viral control (Fig. 6a). While YFV-17D vaccinees are briefly viremic after vaccination^3,5,44,45^, conventional NRG-HIS mice fail to clear the infection (Fig. 1a). Although viremia persisted in NRG-HIS/Flt3LG mice similarly to NRG-HIS mice, viremia in NRGF-HIS/Flt3L mice did not statistically differ from the baseline RNA copy number over time (Fig. 6b; Supplementary Figure 6a). This enhanced control of viral infection correlated with a better survival rate of NRGF-HIS mice (75% versus 50% in NRG-HIS mice survival) over the course of infection (Supplementary Figure 6b). Of note, the observed mortality among the NRGF-HIS/Flt3LG mice cohort (2 out of 8 mice) was not due to lower Flt3LG expression (Supplementary Figure 6c). Five out of the six surviving NRG-HIS/Flt3LG mice, but none of the NRGF-HIS/Flt3L mice, developed significant graft versus host disease (GVHD) by day 20 following infection (Supplementary Figure 6b), which was likely due to the priming of allogeneic T cell responses by the Flt3LG-expanded murine DCs activated by YFV-17D.

The pro-inflammatory cytokine CXCL10 (or IP-10) is one of the most significantly induced cytokines following YFV-17D infection in humans^28,46^. Consistent with these observations, IP-10 serum levels did increase in NRGF-HIS/Flt3LG mice following infection (Fig. 6c). Several other pro-inflammatory cytokines, including MCP-1, IL-6, and IL-18, were also elevated in NRG-HIS/Flt3LG versus NRGF-HIS/Flt3LG mice at specific time points (Supplementary Figure 6d). As these increased cytokine levels did not correlate with an enhanced human immune response in the NRG-HIS/Flt3LG mice, they likely reflected the severe GVHD conditions in NRG-HIS/Flt3LG. Other cytokines, such as IFNγ, IL-23, and GM-CSF, were detected at similar levels in both mouse models (Supplementary Figure 6e).

Although an increase in peripheral CD3+ T cells was observed in both NRG-HIS/Flt3LG and NRGF-HIS/Flt3LG mice, frequencies of CD8+T cells increased in the periphery of only the latter over time (Supplementary Figure 7a, b). These CD8+ T cells upregulated HLA-DR+ and CD38+ T cells, as previously reported in human vaccinees^5^ (Fig. 6d). This distinct phenotypic change of CD8+ T cells and enhanced control of peripheral viral replication in NRGF-HIS/Flt3LG mice also correlated with higher frequencies of multiple myeloid and NK cell subsets in different tissues (Supplementary Figure 7c–e).

### YFV-specific immunity in HLA-expressing NRGF-HIS/Flt3LG mice

**Fig. 7:**
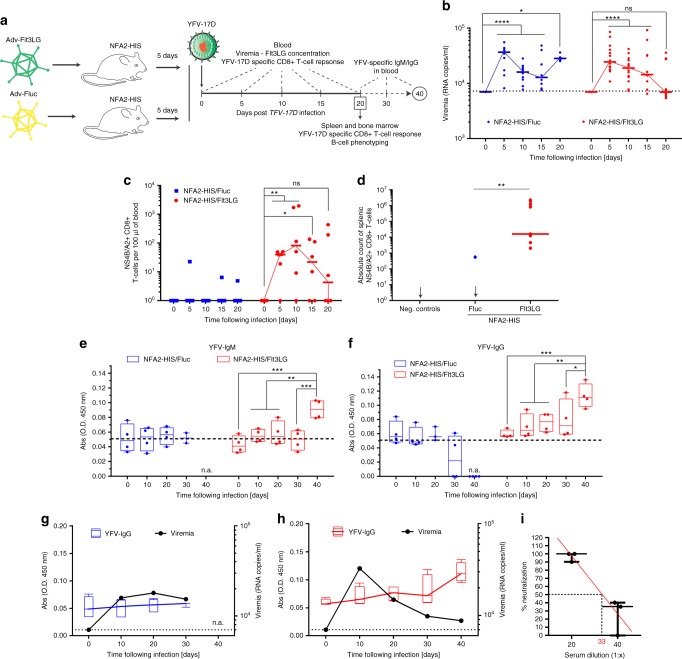
NFA2-HIS/Flt3LG mice mount YFV-specific cellular and humoral response. **a** Schematic representation of the NFA2-HIS mice time course infection experiment. **b** YFV-17D serum viremia in the peripheral blood of NFA2-HIS/Fluc (blue, *n* = 10) and NFA2-HIS/Flt3LG (red, *n* = 14) mice over the course of infection. (+) RNA copies per ml were quantified by RT-qPCR. Limit of detection (dotted line) is shown. Horizontal lines represent median viremia at each time point. *p ≤ 0.05, ****p ≤ 0.0001, ns non-significant (Wilcoxon–Mann–Whitney test). **c** Absolute cell count of peripheral YFV-specific CD8+ T cells (NS4B/A2+) in the blood of NFA2-HIS/Fluc and NFA2-HIS/Flt3LG mice over the course of YFV-17D infection. Cell counts are shown as per 100 μl of total blood. Horizontal lines represent median cell count at each time point (*n* = 4–6). *p ≤ 0.05, **p ≤ 0.01, ns non-significant (Wilcoxon–Mann–Whitney test). **d** Absolute count of YFV-specific CD8+ T cells (NS4B/A2+) in the spleen of NFA2-HIS/Fluc and NFA2-HIS/Flt3LG mice at day 20 post infection. Negative controls represent one non-infected NFA2-HIS/Flt3LG mouse and two infected NRGF-HIS/Flt3LG mice (that do not express HLA-A2). Horizontal lines represent median cell count (*n* = 4–11). **p ≤ 0.01 (Wilcoxon–Mann–Whitney test). **e**, **f** Relative concentration of human anti-YFV-17D IgM (**e**) and IgG (**f**) antibodies in the serum of NFA2-HIS/Fluc (blue, *n* = 4) and NFA2-HIS/Flt3LG mice (red, *n* = 4) over a 6-weeks course of infection. **p ≤ 0.01, ***p ≤ 0.001, ns non-significant (Wilcoxon–Mann–Whitney test). n.a. non applicable as no mice were analyzed at the time of serum collection. **g**, **h** Correlation between YFV-17D viremia (black line) and YFV-IgG relative concentration (colored box and whisker) in the serum of NFA2-HIS/Fluc (**g**) and NFA2-HIS/Flt3LG (**h**) over a 6-weeks course of infection (*n* = 4 per group). Medians in each box and whisker are connected together by a colored line (blue for NFA2-HIS/Fluc and red for NFA2-HIS/Flt3LG). Viremia limit of detection (dotted line) is shown. n.a. non applicable as no mice in the control group were alive  at the time of serum collection. For panels (**e**–**h**), bounds of box and whiskers represent the min-to-max absorbance value at each time point. Medians are indicated in each box as center line. **i** Quantification of YFV-neutralizing activity in the serum of NFA2-NRGF-HIS/Flt3LG mice. Serum neutralizing activity is represented as percentage of YFV-17D infection inhibition (% neutralization). Medians with ranges (min-to-max percentage of neutralization) for both serum dilution are shown (*n* = 3). A linear regression (red line) of the average neutralization activity is shown and was used to determine the median neutralization titer (50% inhibition, red number on the *x*-axis)

Since NRGF-HS/Flt3LG mice did control YFV-17D infection, we analyzed the virus-specific CD8+ T cell response to ascertain its similarity to that of human vaccinees^3,5^. We intercrossed NRG-A2 mice with NRGF mice, yielding NRGF mice expressing HLA-A2*0201 (NFA2 mice). NFA2-HIS mice were then injected with either Adv-Fluc or Adv-Flt3LG 5 days prior to YFV-17D infection (Fig. 7a). Consistent with our previous findings in NRG-HIS/Flt3LG and NRGF-HIS/Flt3LG mice (Fig. 6b), viral replication was better controlled in NFA2-HIS/Flt3LG mice compared to NFA2-HIS/Fluc mice over time (Fig. 7b). Serum viremia in NFA2-HIS/Flt3LG mice peaked between days 5 and 10 post infection with the infection ultimately cleared by day 20 (Fig. 7b). These kinetics mimic those observed in human vaccinees with detectable viremia^3,5,47^. Lower viremia correlated with better survival of the NFA2-HIS/Flt3LG mice (75% versus 30%) over the 3 weeks of infection (Supplementary Figure 8a). Neither cohort developed GVHD. We also found no correlation between survival and differential human Flt3LG concentration in the serum of surviving versus non-surviving NFA2-HIS/Flt3LG mice (Supplementary Figure 8b).

YFV NS4B-tetramer+ CD8+ T cells were readily detectable in the blood of infected NFA2-HIS/Flt3LG mice but not NFA2-HIS/Fluc mice (Fig. 7c; Supplementary Figure 8c). Viremia and the frequencies of YFV-specific CD8+ T cells followed similar kinetics in the peripheral blood, suggesting an important role for CD8+ T cells in YFV-17D infection control and clearance as previously demonstrated in human vaccinees^47^. Consistent with previously reported YFV-specific CD8+ T cell phenotypes from human vaccines^3^, a significant fraction of YFV NS4B-tetramer+ CD8+ T cells proliferated, as indicated by their Ki67 expression, and acquired an HLA-DR+/CD38+ effector phenotype (Supplementary Figure 8d, e). In contrast, NS4B-tetramer negative CD8+ T cells did not demonstrate upregulated Ki-67 expression upon YFV-17D infection (Supplementary Figure 8f).

Although the frequency of antigen-specific CD8+ T cells statistically decreased to background levels in the blood of NFA2-HIS/Flt3LG mice, YFV-specific CD8+ T cells were readily detectable in the spleen by day 20 post infection but were not detectable in the spleen of NFA2-HIS/Fluc mice (Fig. 7d, Supplementary Figure 8g). Absolute cell count and phenotyping of splenic YFV-specific CD8+ T cells showed that these cells mostly expressed HLA-DR and CD38 at day 20 post infection (Supplementary Figure 8g–i). They also did not show preferential expression of Ki67, suggesting a switch toward a memory phenotype. Future studies will be aimed at accurately delineating the different phenotypes of these antigen-specific cells.

Given the important correlation between the induction of a T cell-specific response and the clearance of YFV-17D infection in the periphery, we conducted a T cell depletion experiment. Prior to YFV-17D infection and 5 days post infection, NFA2-HIS/Flt3LG mice were treated with anti-CD4 (α-CD4) or anti-CD8 (α-CD8) antibodies (*n* = 4 per group), which have previously been used to deplete CD4+ and CD8+ T cells, respectively, in humanized mice^7,9^ (Supplementary Figure 9a). Peripheral CD4+ and CD8+ T cells were efficiently depleted in α-CD4 and α-CD8-treated NFA2-HIS/Flt3LG mice, respectively (Supplementary Figure 9b), prior to infection. In the spleen, where T cells are more abundant than in the peripheral blood, we observed a more than ten-fold reduction in the number of CD4+ T cells in α-CD4-treated mice and a more than 1000-fold reduction in the number of CD8+ T cells in α-CD8-treated mice (Supplementary Figure 9c). Importantly, the counts of myeloid cell populations were unaffected in either condition (Supplementary Figure 9d). Upon T cell depletion, only α-CD8-treated mice exhibited significant mortality upon infection (50% survival) (Supplementary Figure 9e). However, both α-CD4-treated and α-CD8-treated mice were unable to clear viral infection in the periphery (Supplementary Figure 9f). Thus, these results suggest that both CD4+ and CD8+ T cells are important for controlling infection in peripheral blood and that CD8+ T cells are likely early regulators of such control. Additionally, these results are further evidence that the clearance of YFV-17D infection in NFA2-HIS/Flt3LG mice is human-, and not murine-, mediated.

Seroconversion in human vaccinees is the hallmark of YFV-17D potent immunogenicity^48^. Hence, determined whether the enhanced control of infection and T cell-specific response were also associated with an improved humoral immune response. We assessed YFV-specific antibody concentrations in the sera of NFA2-HIS/Fluc (*n* = 4) and NFA2-HIS/Flt3LG (*n* = 4) mice over 6-weeks following infection. We detected a significant increase in YFV-specific IgM and IgG in the serum of the four NFA2-HIS/Flt3LG mice at day 40 post infection (Fig. 7e, f; Supplementary Figure 10a). In contrast, YFV-specific antibodies were not detected in the serum of NFA2-HIS/Fluc mice during the first 30 days following infection, and none of these mice survived till the final experimental end-point (day 40 post infection). Notably, we found a strong negative correlation between viremia level and YFV-specific IgG concentration in the blood of NFA2-HIS/Flt3LG mice but not in NFA2-HIS/Fluc mice (Fig. 7g, h). Consistently, the serum of three NFA2-HIS/Flt3LG mice at day 40 post infection neutralized YFV-17D in vitro (median neutralizing titer: 1:33) (Fig. 7i). NFA2-HIS/Flt3LG mice also displayed a significantly enhanced frequency of multiple B cell subpopulations at day 20 post infection in comparison to NFA2-HIS/Fluc mice (Supplementary Figure 10b, c). Specifically, frequencies of follicular B cells, transitional B cells, class-switched memory B cells, or plasmablasts were higher in NFA2-HIS/Flt3LG mice, suggesting these subpopulations proliferate and differentiate better in response to YFV-17D infection in this model.

### Enhanced YFV-17D immunity associates with superior HIS complexity

Finally, we employed Seq-Well^49^, a recently developed platform for massively parallel single-cell RNA-Seq (scRNA-Seq), to delineate at the greatest possible resolution the cellular composition of the engrafted HIS that correlated with a superior human immune response to YFV-17D. We isolated splenocytes from two NRG-HIS mice and two NFA2-HIS/Flt3LG mice at 6-weeks post YFV-17D infection and sorted these cells by human CD45 (hCD45) or human CD33 (hCD33) expression (see also Supplementary Note 1).

**Fig. 8:**
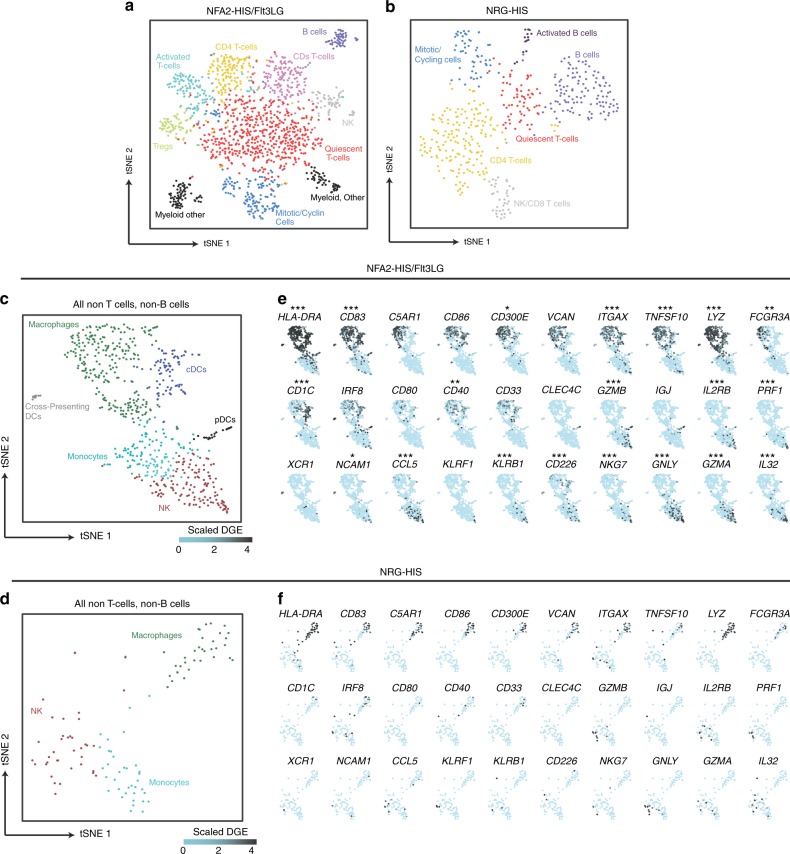
Improved human immune system complexity in NFA2-HIS/Flt3LG. **a**, **b** 1297 and 457 single cells from the CD45+ compartment in NFA2-HIS/Flt3LG (**a**) and NRG-HIS (**b**) mouse spleens respectively, 6 weeks post infection. Single cells are plotted using t-stochastic neighbor embedding (tSNE). Significant clusters were defined using a shared nearest neighbor modularity based clustering algorithm^67^. Clusters identified by defining significantly differentially expressed genes in each cluster using a likelihood ratio test for single-cell gene expression and annotating by literature-supported gene expression programs or subpopulation defining genes (see Methods section). **c**, **d** CD3-CD79-TCR-BCR- single cells from both CD45+ and CD33+ compartments in spleens from two NFA2-HIS/Flt3LG (**c**) and NRG-HIS (**d**) mice are plotted as a tSNE. Clustering and annotation are completed as described in (**a**, **b**). **e**, **f** Cluster-defining genes over CD3-CD79-TCR-BCR- single cells from NFA2-HIS/Flt3LG (**e**) and NRG-HIS (**f**) mice are represented as a projected color scale on the tSNE calculated in (**c**) and (**d**), respectively. Scaled digital gene expression is represented as a color map from light blue (low gene expression) to black (high gene expression), and these values are projected onto the single cell point. The expression of these genes in NFA2-HIS/Flt3LG mice (**e**) was compared to the expression in NRG-HIS mice (**f**), and significantly differentially expressed genes are shown in (**e**) (*p ≤ 0.05, ***p ≤ 0.001; Benjamini–Hochberg adjusted *p*-value). Tregs regulatory T cells, NK natural killer cells, DCs dendritic cells, cDCs conventional dendritic cells, pDCs plasmacytoid dendritic cells

We ran parallel Seq-Well arrays for each sorted population, enabling both unbiased characterization of the relative abundances of all lymphocytes, as well as a deeper examination of the cellular diversity within the myeloid compartment. First, we examined the cell types identified in hCD45+single cells in NFA2-HIS/Flt3LG (Fig. 8a) compared to NRG-HIS (Fig. 8b) mice. NFA2-HIS/Fl3LG splenic CD45+ cells showed a higher diversity of well-resolved subpopulations of activated and differentiated cytotoxic lymphocytes, T cells expressing known activation and memory markers (*CD27*, *CCR7*, *STAT1*, *CD40LG*), and regulatory T cells (distinguished by high expression of *FOXP3* and *CTLA4*). The abundance of myeloid and NK cells within the hCD45+ samples was significantly higher in NFA2-HIS/Flt3LG mice, and we were unable to resolve a distinct cluster of myeloid cells from the NRG-HIS populations when clustered alone.

To compare more directly the myeloid compartments between the NRG-HIS mice and the NFA2-HIS mice, we combined both hCD33+ and hCD45+ samples from either the NFA2-HIS mice (Fig. 8c) or NRG-HIS mice (Fig. 8d) and computationally gated out all T cells and B cells by expression of TCR- or BCR-related genes (full list in Methods section). In NFA2-HIS/Flt3LG mice, we identified six distinct clusters of cell types corresponding to subpopulations of NK cells, monocytes, macrophages, cDCs, pDCs, and cross-presenting DCs (full cluster-defining genes in Supplementary Data 2). We also directly compared the expression of the top subpopulation-defining genes in NFA2-HIS/Flt3LG non-T, non-B single cells (Fig. 8e) with the corresponding single cell gate in NRG-HIS mice (Fig. 8f). This analysis revealed significant up-regulation of a broad range of myeloid and NK cell functional and activation markers (such as *HLA-DRA*, *CD83*, and *CD40* for cDCs, and *CCL5, GNLY, GZMA, PRF1*, and *CD226* for NK cells) in NFA2-HIS/Flt3LG mice over NRG-HIS mice.

**Fig. 9:**
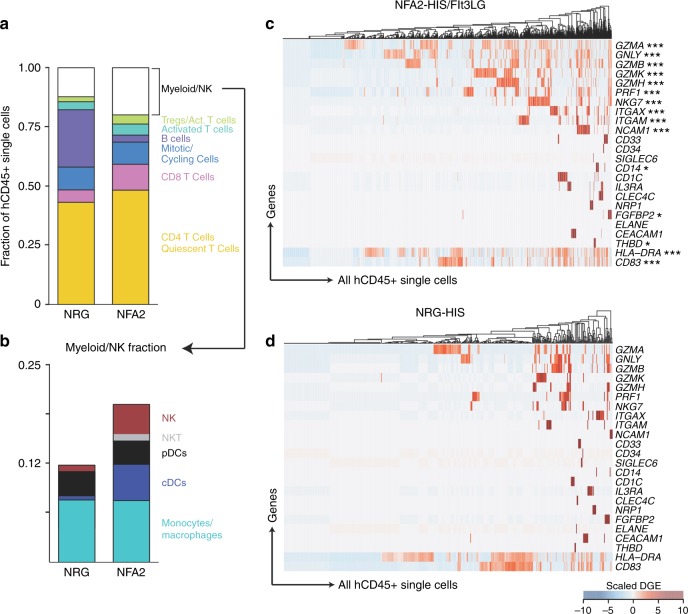
ScRNA-Seq-based measurement of the myeloid and NK cell engraftment. **a**, **b** Chart bart displaying the fraction of human cell subsets within the hCD45+ compartment in both NRG-HIS and NFA2-HIS/Flt3LG mouse spleens (**a**). The fraction of multiple myeloid and NK subsets is highlighted within a second chart bart (**b**). Cell subsets are colored as in Fig. 8a, c. **c**, **d** Heatmap of all hCD45+ single cells in NFA2-HIS/Flt3LG (**c**) and NRG-HIS (**d**) mice over myeloid, DC, NK, and granulocyte subpopulation-defining genes. Differentially expressed genes between NRG-HIS and NFA2-HIS/Flt3LG mice are shown in (**c**) (*p ≤ 0.05, **p ≤ 0.01, ***p ≤ 0.001; Benjamini–Hochberg adjusted *p*-values). Tregs regulatory T cells, NK natural killer cells, NKT natural killer T cells, DCs dendritic cells, cDCs conventional dendritic cells, pDCs plasmacytoid dendritic cells

When analyzed alone, hCD45+ NRG-HIS single cells did not yield a distinct subpopulation that could be annotated as a myeloid type cluster. However, when these cells were clustered in concert with abundant myeloid cell types from the NFA2-HIS mice, we could resolve these cells distinctly. Through this analysis, we confirmed superior frequencies of DCs, NK cells and NKT cells in NFA2-HIS/Flt3LG mice in comparison to NRG-HIS mice (Fig. 9a, b).

Finally, we tested differential expression between hCD45+ single cells in both mouse models over a curated list of lineage and cell-type relevant genes (Fig. 9c, d). This analysis revealed significantly higher expression of functional markers of activated NK and T cells (such as *NKG7, PRF1*, and *GZMA)*, as well as higher abundance of functional mature myeloid cells (notably via *ITGAM*, *ITGAX*, *CD14*, or *CD83)* in NFA2-HIS/Flt3LG mice.

Altogether, these data provide an in-depth view of the cellular composition of the HIS in conventional and second-generation humanized mice. They also highlight the enhanced engraftment and functionality of the myeloid and NK cell compartment in NFA2-HIS/Flt3LG mice, which is likely critical in promoting an enhanced transcriptomic, cellular, and humoral response to YFV-17D. A more complete description of our experimental design and our scRNA-seq data can be found in Supplementary Note 1, associated with Supplementary Figure 10d, Supplementary Figure 11, Supplementary Figure 12, and Supplementary Data 3.

## Discussion

Understanding the pathogenesis and immune responses elicited by human-tropic pathogens presents considerable challenges. Over the past decades, humanized mice have proven susceptible to a large number of human-tropic pathogens^6,8,17,27,50,51^ and have emerged as valuable platforms to model human-specific infectious processes in vivo. Despite the fact that multiple studies have reported that humanized mice can develop immunological features resembling those of humans, commonly used humanized mouse models still mount an imperfect human immune response^7,9,16,52^, and it remains incompletely defined how specific refinements of xenorecipient strains and/or humanization protocols impact immunity. To guide a more directed approach to improving future generations of humanized mice, objective metrics are needed to facilitate direct comparisons with clinical data. YFV-17D is a highly potent human vaccine that induces a strong, polyfunctional immune response^3,28,44^ as well as long-lasting protective immunity (reviewed in ref. ^26^). The human immune response to YFV-17D has been intensively studied in the peripheral blood of human vaccinees^3,5,28,44^, and transcriptomic signatures in human PBMCs following YFV-17D immunogenicity have been delineated^28,44^. These features highlight YFV-17D human immunity as a powerful comparator for evaluating immune responses in humanized mice.

Here, we provide a comprehensive and quantitative comparison of human clinical data with equivalent data sets from conventional and exemplary second-generation humanized mouse models. By probing the specific cellular, humoral, and transcriptomic response to YFV-17D in different humanized mouse models, we examined how a correlation index, built to reflect the degree of overlap between the transcriptome of a given humanized mouse model and that of human vaccinees upon YFV-17D infection, associated with the induction of YFV-specific immunity and viral clearance within a given model.

Despite high levels of human engraftment, the human immune response to YFV-17D in NRG-HIS mice was weak and associated with a low correlation index. In contrast, enhancement of the NK and myeloid cell compartments in NRGF-HIS associated with a significantly higher correlation index in comparison to conventional humanized mice, which translated into the induction of a specific cellular and humoral response against YFV-17D. Hence, our study shows that the transcriptomic correlation index represents a powerful and accurate proxy for assessing the quality of the human immune response in these—and importantly, other—models.

Numerous alternative humanization strategies have been realized in e.g., so-called BLT or MISTRG models^17,23^. The experimental pipeline and data sets provided here will undoubtedly be a valuable resource to objectively evaluate these strategies in comparison to others, as well as for the rational development of advanced humanized mice models and improved modeling of human disease in vivo. Refinement strategies could include the expression of additional human orthologs of non-redundant cytokines, which exhibit limited biological cross-reactivity in order to foster development of other underrepresented human immune cell types. Currently, most studies employ naive, i.e., antigen-inexperienced, mice, which does not take into account how immunological history shapes immunity. Thus, exposing mice to multiple other vaccines prior to yellow fever vaccination or infection with another pathogen may be a valuable approach to further enhance immunity. Co-engraftment of NFA2 or other xenorecipient strains with additional HSC donor-matched human tissues, such as liver, thymus, and/or lymph nodes, could also significantly augment the immune response. Such co-engraftments could enhance T and B cell selection, intra-hepatic T cell priming^53^ as well as liver-mediated secretion of key human-immune components^54^. Finally, engraftment of second-generation humanized mice with a human-like microbiome represents another valuable approach to enhance immunity as recently suggested^55^.

Taken together, our study demonstrates that correlation of transcriptomic signatures provide a relevant and valuable path toward the rational refinement of humanized mouse models. Such an approach opens avenues for more accurate characterization of human–pathogen interaction events in vivo, for the development of innovative immunotherapy and vaccine strategies, as well as for the modeling of relevant (patho-) physiological processes and auto-immune diseases.

## Methods

### Cell lines and antibodies

HEK293, HEK293T cells (both American Type Culture Collection, Manassas, VA) and Huh7.5 (kindly provided by Charles Rice, The Rockefeller University) were grown in Dulbecco’s modified Eagle’s medium (DMEM) supplemented with 10% heat inactivated fetal bovine serum (Thermo Scientific, Waltham, MA, USA) and 1% Penicillin Streptomycin (Thermo Scientific, Waltham, MA, USA). The following anti-mouse Abs were used: From Biolegends (San Diego, CA, USA): CD45-PE-Cy7 clone 30-F11 (dilution 1/100), CD3-PerCP-Cy5.5 clone 17A2 (dilution 1/100), Ly6G/Gr1-PerCP-Cy5.5 HK1.4 (dilution 1/50); From BD Biosciences (San Jose, CA, USA): CD11c-allophycocyanin clone HL3 (dilution 1/50); From eBiosciences/Thermo Fisher scientific (San Diego, CA, USA): CD11b-allophycocyanin-eFluor 780 clone M1/70 (dilution 1/100), F4/80-PE clone BM8 (dilution 1/100), CD317-Alexa Fluor 488 clone eBio927 (dilution 1/50), TER-119-PerCP-Cy5.5 clone TER-119 (dilution 1/50), CD19-PerCP-Cy5.5 clone 1D3 (dilution 1/100), NK1.1-PerCP-Cy5.5 clone PK136 (dilution 1/100). The following anti-human Abs were used: From BD Biosciences (San Jose, CA, USA): CD45-V500 clone HI30 (dilution 1/100), CD19-allophycocyanin-Cy7 clone SJ25C1 (dilution 1/100), CD4-allophycocyanin clone RPA-T4 (dilution 1/100), CD4-Alexa Fluor 700 clone RPA-T4 (dilution 1/100); CD8-FITC clone G42-8 (dilution 1/100), IgG-FITC clone G18-145 (dilution 1/25), CD138-BV421 clone MI15 (dilution 1/50); From Life Technologies, Invitrogen (Carlsbad, CA, USA); CD3-PE-Cy5 clone 7D6 (dilution 1/100); Ki-67-PE clone 20Raj1 (dilution 1/50); From Biolegends: CD56-allophycocyanin-Cy7 clone HCD56 (dilution 1/100), CD45RA-Alexa Fluor 700 clone HI100 (dilution 1/100); From Thermo-scientific/eBiosciences (Waltham, MA, USA/San Diego, CA, USA): CD3-allophycocyanin-eFluor780 clone UCHT1 (dilution 1/100), CD33-PerCP-Cy5.5 clone WM53 (dilution 1/100), CD14-PE-eFluor610 clone 61D3 (dilution 1/50), CD11c-Alexa Fluor 700 clone 3.9 (dilution 1/50), CD68-PE clone Y1/82A (dilution 1/50), HLA-DR-eFluor450 clone L243 (dilution 1/100), CD279/PD-1-allophycocyanin-eFluor780 clone J43 (dilution 1/50), CD38-PE-eFluor610 clone HIT2 (dilution 1/50), CD197/CCR7-PE clone 3D12 (dilution 1/50), CD1c/BDCA-1-FITC clone L161 (dilution 1/50), CD123-eFluor450 clone 6H6 (dilution 1/50), CD19-PE-eFluor610 clone HIB19 (dilution 1/100), CD33-PE clone WM53 (dilution 1/100), CD56-FITC clone TULY56 (dilution 1/50), CD27-PE clone O323 (dilution 1/50), CD28-PE-eFluor610 clone CD28.2 (dilution 1/50), CD127-eFluor450 clone A7R34 (dilution 1/50), CD20-Alexa Fluor 700 clone 2H7 (dilution 1/50), CD24-eFluor450 clone eBioSN3 (dilution 1/50), IgD-PerCP-eFluor710 clone IA6-2 (dilution 1/50), IgM-FITC clone SA-DA4 (dilution 1/50); From Milteny Biotec (Cambridge, MA, USA): CD303/BDCA-2-allophycocyanin clone AC144 (dilution 1/50), CD141/BDCA-3-FITC clone REA674 (dilution 1/50); From Novus Biological (Littleton, CO, USA): CD66b-Alexa Fluor 700 clone G10F5 (dilution 1/50); From BioXCell (West Lebanon, NH, USA): CD8α clone OKT-8 (100 µg per mouse and per injection), CD4 clone OKT-4 (100 µg per mouse and per injection).

### Adenovirus constructs

Adenoviral constructs encoding for the firefly luciferase (Fluc) or Flt3LG were created using the AdEasy Adenoviral Vector System (Agilent Technologies, Santa Clara, CA, USA) according to the manufacturer’s instructions. Briefly, Fluc and human Flt3LG cDNA coding sequence were cloned following restriction digest (KpnI-XhoI and KpnI-EcorV, respectively) into pShuttle TRE-pAdEasy, leading pShuttle TRE-pAdEasy-Fluc and pShuttle TRE-pAdEasy-Flt3LG, respectively. Recombinant pShuttle-CMV plasmids were linearized with PmeI and ligated to pAdEasy by homologous recombination followed by electroporation into BJ5183 cells (Agilent Technologies, Santa Clara, CA, USA). Recombinant pShuttle-pAdEasy constructs were identified by PacI restriction analysis. All plasmid constructs were verified by DNA sequencing.

### Generation of recombinant adenoviruses

Adenoviral stocks were generated as previously described^56^. Briefly, adenoviral constructs were transfected into HEK293 cells (American Type Culture Collection, Manassas, VA) using the calcium-phosphate method. Transfected cultures were maintained until cells exhibited full cytopathic effect (CPE), then harvested and freeze-thawed. Supernatants were serially passaged two more times with harvest at full CPE and freeze thaw was then performed. For virus purification, cell pellets were resuspended in 0.01 M sodium phosphate buffer pH 7.2 and lysed in 5% sodium-deoxycholate, followed by DNase I digestion. Lysates were centrifuged and the supernatant was layered onto a 1.2–1.46 g ml^−1^ CsCl gradient, then spun at 95,389*g* on a Beckman Optima 100K-Ultra centrifuge using an SW28 spinning-bucket rotor (Beckman-Coulter, Brea, CA, USA). Adenovirus bands were isolated and further purified on a second CsCl gradient using an SW41Ti spinning-bucket rotor. Resulting purified adenoviral bands were isolated using a 18.5G needle and twice-dialysed against 4% sucrose. Adenovirus concentrations were measured by reading the OD_260_ on a FLUOstar Omega plate reader (BMG Labtech, Ortenberg, Germany). Adenovirus stocks were aliquoted and stored at −80 °C.

### Isolation of human CD34+ HSC

All experiments were performed with authorization from the Institutional Review Board and the IACUC at Princeton University. Human fetal livers (16–22 weeks of gestational age) were procured from Advanced Bioscience Resources (ABR), Inc. (Alameda, CA). Fetal liver was homogenized and incubated in digestion medium (HBSS) with 0.1% collagenase IV (Sigma-Aldrich, Darmstadt, Germany), 40 mM HEPES, 2 M CaCl_2_, and 2 U/ml DNAse I (Roche, Basel, Switzerland) for 30 min at 37 °C. Human CD34+ HSC were isolated using a CD34+ HSC isolation kit (Stem Cell Technologies, Vancouver, British Columbia, Canada) according to the manufacturer’ protocol. Purification of human CD34+ cells were assessed by quantifying by flow cytometry using an anti-human CD34+-FITC antibody (dilution 1/100, clone 581, BD Biosciences, Franklin Lakes, NJ). Expression of human CD90, CD38, CD45RA was assessed among the CD34+ population.

### Construction of NRGF and NFA2 mice

NRG-Flk2−/− mice were generated by backcrossing mice harboring a Flk2 null allele (*Flt3*^*tm1Irl*^, kindly provided by Ihor Lemishka, Mount Sinai School of Medicine, NY) for 10 generations to NOD *Rag1*^−/−^ *IL2Rg*^*null*^ mice (NOD.Cg-*Rag1*^*tm1Mom*^*Il2rg*^*tm1Wjl*/SzJ^, obtained from the Jackson Laboratory, catalog number 007799). At each generation, the presence of each mutant allele was confirmed with allele-specific primers. Resultant NRG-Flk2+/− were intercrossed to produce NRG-Flk2−/− mice. NRG-HLA-A2*0201 were generated by intercrossing NSG-A2*0201 (NOD.Cg-*Prkdc*^*scid*^*IL2rg*^*tmlWjl*^*/Sz* Tg(HLA-A2.1)1Eng/Sz)^7,9^ with NRG mice. The absence of the SCID mutation and the presence of the *Rag1, IL2Rγ* null alleles and the A2 transgene was determined by PCR. To generated NRG-Flk2−/− HLA-A*0201 (NFA2) mice, NRGF and NRG-A2 mice were intercrossed. NRG, NRG-A2, NRGF, and NFA2 mice were maintained at the Laboratory Animal Resource Center at Princeton University.

All animal experiments described in this study were performed in accordance with protocols (number 1930) that were reviewed and approved by the Institutional Animal Care and Use and Committee of Princeton University.

### Generation of human immune system-engrafted mice

1–5 days old xenorecipient mice were irradiated with 300 cGy and 1.5–2 × 10^5^ human CD34+ HSC were injected intrahepatically 4–6 h after irradiation. Male and female mice transplanted with CD34+ HSC derived from various human donors were used in this study.

### Mouse injections and blood collections

4–8-month-old NRG-HIS, NRGF-HIS, and NFA2-HIS were infected through intravenous injection in the tail with 10^10^ recombinant AdV-Flt3LG or 10^11^ recombinant AdV-Fluc particles and/or with 10^6^ YFV-17D p.f.u., resuspended in 200 µl of PBS. For experiments involving pre-injection of AdV-Fluc or AdV-Flt3LG prior YFV-17D infection, YFV-17D infection was always performed 5 days following AdV-injection. 200 µl of blood were collected through submandibular bleeding at the indicated time-points. Serum was separated from blood cells by centrifugation (10 min, 3500 rpm at room temperature) for further quantification of serum viremia. For assessment of immune cell expansion and time course experiments, all the infected humanized mice displayed a level of peripheral humanization ranging from 40 to 60% human CD45+ out of total CD45+ cells. For assessment of the YFV-17D transcriptomic signature, all humanized mice displayed level of peripheral humanization ranging from 50 to 80% human CD45+ out of total CD45+ cells.

### Monitoring of clinical symptoms and manifestations

Clinical manifestations of disease were monitored daily and signs of clinical disease progression recorded through weight and clinical scoring. All mice succumbing to YFV-17D infection developed severe weight loss and displayed several signs of disease such as posture hunched, trembling, appearance with ruffled fur and rear leg paralysis (at later stage of disease). GVHD was determined by the presence of the following clinical symptoms: severe hair loss on a significant portion of the body (with visible naked skin), skin rash, and skin inflammation.

### Quantification of human FLT3LG concentration in mouse serum

Human Flt3LG concentrations were measured using an in-house sandwich ELISA. A rabbit polyclonal capture (Abcam, Cambridge, MA, USA) antibody was coated overnight into a 96 well-plate (Thermo Scientific, Waltham, MA, USA) at a concentration of 1:500. Following incubation with a blocking-buffer (SuperBlock™ Blocking Buffer; Thermo Scientific, Waltham, MA, USA), mouse serums were incubated at multiple dilution (1:10, 1:100, 1:1000). Captured human Flt3LG was then detected using a biotin-conjugated rabbit polyclonal detection antibody (Abcam, Cambridge, MA, USA) and a streptavidin-conjugated horseradish peroxidase (HRP, Abcam, Cambridge, MA, USA) antibody. A soluble recombinant human Flt3LG (initial concentration: 100 ng/ml) was used to determine a standard concentration curve. Optical density signals at 450 nm were then assessed using a TriStar Multimode Microplate reader (Berthold Technologies GmbH & Co. KG, Bad Wildbad, Germany).

### Quantification of YFV-specific antibodies in mouse serum

Relative concentration of human YFV-17D-specific IgM and IgG was measured by homemade sandwich enzyme-linked immuno-sorbent assay (ELISA). 5000 YFV-17D infectious particles were coated overnight in a 96-well plate (Thermo Scientific, Waltham, MA, USA). Following incubation with a blocking-buffer (SuperBlock™ Blocking Buffer; Thermo Scientific, Waltham, MA, USA), mouse serums were incubated at 1:20 and 1:40 dilution. A serum sample from an anonymous human vaccinee was diluted from 1:10 to 1:160 in a two-fold dilution manner, and used as a positive control. Captured IgG and IgM were then detected using anti-human IgG (Thermo Scientific, Waltham, MA, USA; Clone HP6017) or IgM (Thermo Scientific, Waltham, MA, USA; Clone HP6083) horse peroxidase antibodies. Optical density signals at 450 nm were then assessed using a TriStar Multimode Microplate reader (Berthold Technologies GmbH & Co. KG, Bad Wildbad, Germany).

### YFV-17D infectious clone and in vitro transcription

pACNR-YFV-17D low-copy number backbone (kindly provided by Charles Rice, The Rockefeller University, NY) was transformed and amplified using low recombination NEB 5-alpha high efficiency competent *Escherichia coli* (New England Biolabs, Ipswich, MA, USA). Transformed bacteria were incubated in LB+ 50 μg/ml Ampicillin (Sigma-Aldrich, Darmstadt, Germany) overnight at 30 °C under shaking at 205 rpm. Plasmid cDNA was purified using E.Z.N.A. Endonuclease free Maxiprep Kit (Omega, Norcross, GA, USA), ethanol precipitated and linearized using Afl-II restriction enzyme. Following concentration of linearized DNA by ethanol precipitation, viral RNA was transcribed from 1 µg of linear template using mMESSAGE mMACHINE SP6 kit (Ambion, Foster City, CA, USA) according to manufacturer’s instructions.

### Electroporation and production of YFV-17D stocks

Huh-7.5 cells were washed twice with Opti-MEM Gluta-Max-1 reduced serum media (Life Technologies, Invitrogen, Carlsbad, CA, USA) and resuspended at a concentration of 1.5 × 10^7^ cells/ml in Opti-MEM. 2 µg of viral RNA was mixed with 0.4 ml of cell suspension and immediately pulsed in a 2 mm cuvette using a BTX ElectroSquare Porator ECM 830 (860 V, 99 µs, five pulses) (BTX, Holliston, MA, USA). Electroporated cells were incubated at room temperature for 10 min and series of 3 consecutive electroporations (for a total 6 µg for 1.8 × 10^7^ cells) were then dripped into 25 ml (P150 culture dish) of media. At 24 h post electroporation, media was changed and replaced by low-serum concentration DMEM (1% FBS). Virus was collected at 48 and 72 h post electroporation. At 72 h post electroporation, virus was polled and concentrated 40–100-fold using a Millipore 10,000 MWCO spin filter columns (Merck Millipore, Darmstadt, Germany) on the last day of collection (3000g, 20 min). Viral titer was then assessed using a plaque forming unit assay.

### Titration of viral stocks and YFV-17D in vitro infections

To determine the viral titer of the YFV-17D stock, 2.5 × 10^5^ Huh7.5 cells were seeded per well in 6-well plates 24 h post infection. Serial dilution from 10^−3^ to 10^−12^ of the viral stocks were prepared, and 2 ml of each dilution were incubated with Huh7.5 for 6 h at 37 °C. At 6 h post infection, media were replaced by a fresh methocell solution (DMEM, 10% FBS, 1% Methylcellulose). Four days post infection, cells were washed with PBS, fixed with 100% ethanol for 25 min and stained with 0.1% (w/vol) crystal violet. The number of plaques (plaque forming unit, p.f.u.) for each dilution was then determined.

### Organ collection and isolation of immune cells

Blood (200 µl) was collected through submandibular bleeding and transferred into EDTA capillary collection tubes (Microvette 600 K3E, Sarstedt, Nümbrecht, Germany). Cells were separated from plasma through centrifugation, and red blood cells were lysed with 1× lysis buffer (BD Pharm Lyse, BD Biosciences, San Jose, CA, USA) for 15 min at room temperature in the dark. Following lysis and quenching with 10% (v/v) FBS DMEM media, blood cells were then washed twice with a 1% (v/v) FBS–PBS solution before staining. At the indicated endpoints, mice were euthanized via exsanguination under ketamine/xylazine anesthesia. To generate splenocyte single cell suspensions, spleens were collected and individually placed in 15 ml of serum-free DMEM. Spleens were then transferred in a 6 cm dish, dissociated using a razor blade and digested (0.1% collagenase, Sigma-Aldrich, Darmstadt, Germany; 40 mM HEPES; 2 mM CaCl_2_; 2 U/ml DNase1, HBSS, Life Technologies, Invitrogen, Carlsbad, CA, USA) for 30 min at 37 °C. Following quenching with 10% (v/v) FBS-DMEM media, splenocytes were strained through a 100 µm cell strainer and washed with 10% (v/v) FBS-DMEM twice. Splenocytes were then centrifuged and lysed with 1× lysis buffer (BD Pharm Lyse, BD Biosciences, San Jose, CA, USA) for 15 min at room temperature in the dark. Cells were then washed twice with a 1% (v/v) FBS–PBS solution and counted prior to staining. For bone marrow-derived cell isolation, femurs and tibiae of the mice were flushed with PBS (Life Technologies, Invitrogen, Carlsbad, CA, USA) in a 10 cm dish. Cells were then centrifuged and lysed with 1× lysis buffer (BD Pharm Lyse, BD Biosciences, San Jose, CA, USA) for 10 min at room temperature in the dark. Cells were then washed twice with a 1% (v/v) FBS–PBS solution and counted prior to staining.

### YFV-17D single-step RT-quantitative real time PCR

Viral RNA was isolated from mouse serum using the ZR Viral RNA Kit (Zymo, Irvine, CA, USA) according to manufacturer’s instructions. Viral RNA was quantified using single-step RT-quantitative real-time PCR (SuperScript® III Platinum® One-Step qRT-PCR Kit, Life Technologies, Invitrogen, Carlsbad, CA, USA) with primers and TaqMan probes targeting a conserved region of the 5′UTR of the 17D genome. Single-step RT***-***qPCR was accomplished in a StepOnePlus Real-Time PCR System (Applied Biosystems) using the following thermal cycling: 52 °C for 15 min, denaturation at 94 °C for 2 min, 40 cycles of denaturation at 94 °C for 15 s, annealing at 55 °C for 20 s, and elongation at 68 °C for 20 s. A cDNA sequence coding for the 5′UTR was in vitro transcribed and used as standard for the absolute quantification of viral RNA. The primers used are as follow: YFV-17D sense 1, GCTAATTGAGGTGCATTGGTCTGC; YFV-17D sense 2, GCTAATTGAGGTGTATTGGTCTGC; YFV-17D antisense 1, CTGCTAATCGCTCAACGAACG; YFV-17D antisense 2, CTGCTAATCGCTCAAAGAACG, YFV-17D probe, FAM-ATCGAGTTGCTAGGCAATAAACAC-BHQ.

### Antibody staining and flow-cytometry analysis

2–4 × 10^6^ PBMCs, splenocytes, or bone marrow cells of human or murine origins were isolated as described above and stained for 1 h at 4 °C in the dark with the appropriate antibody cocktail. Following washing (1% (v/v) FBS in PBS), cells were fixed with fixation buffer (1% (v/v) FBS, 4% (w/v) PFA in PBS) for 30 min at 4 °C in the dark. Flowcytometric analysis was performed using an LSRII Flow Cytometer (BD Biosciences, San Jose, CA, USA). Flow cytometry data were analyzed using FlowJo software (TreeStar, Ashland, OR). Chimerism of all humanized mice model was assessed prior each experiment by quantifying the following human populations: Human CD45+, human CD45+ murine CD45−; T-cells, CD45+ CD3+; CD4+ T cells, CD45+ CD3+ CD4+; CD8+ T cells, CD45+ CD3+ CD8+; CD45+ CD16+ leukocytes; B-cells, CD45+ CD19; conventional dendritic cells, CD45+ CD11c+; NK/NKT cells, CD45+ CD56+; Monocytes, CD45+ CD14+. Mouse immune cell subsets were gated as followed: Murine CD45+, Human CD45− Murine CD45+; Conventional dendritic cells, CD45+ CD3− CD19− NK1.1− TER119− Ly-6G/Gr1− CD11c+; Plasmacytoid dendritic cells, CD45+ CD3− CD19− NK1.1− TER119− Ly-6G/Gr1− CD317+; Monocytes, CD45+ CD3− CD19− NK1.1− TER119− Ly-6G/Gr1− CD11b+ CD11c− F4/80−; Macrophages, CD45+ CD3− CD19− NK1.1− TER119− Ly-6G/Gr1− CD11b+ F4/80+. Human immune cell subsets were gated as followed: Human CD45+, human CD45+ murine CD45−; T-cells, CD45+ CD3+; CD4+ T cells, CD45+ CD3+ CD4+; CD8+ T cells, CD45+ CD3+ CD8+; Myeloid cells, CD45+ CD3− CD19− (CD56+) CD33+; Granulocytes, CD45+ CD66b+; B cells, CD45+ CD3− CD19+; Natural Killer cells, CD45+ CD3− (CD19−) CD56+; Natural Killer T cells and γδ T cells, CD45+ CD3+ (CD19−) CD56+; Conventional dendritic cells, CD45+ CD3− CD19− (CD56−) (CD33+) CD11c+ (BDCA1/3+); CD45+ CD3− CD19 CD123+, group composed of monocytes, plasmacytoid dendritic cells, basophils and myeloid precursors; Plasmacytoid dendritic cells, CD45+ CD3− CD19− (CD56−) BDCA-2+ CD123+; Monocytes, CD45+ CD3− CD19− (CD56−) CD14+; Macrophages, CD45+ CD3− CD19− (CD56−) CD68+.

For B-cell phenotyping, human bone marrow-derived CD19+ were analyzed for their expression of CD20, CD24, CD38, CD27, CD138, IgD, IgG and IgM. Sub-populations were qualified as follow: naive B-cells, CD19+ IgD+ CD27−; non-classed switched memory B-cells (NCS memB), CD19+ IgD+ CD27+; Class-switched memory B-cells (CS memB), CD19+ IgD− CD27+ CD20+ IgM−; Plasmablast cells, CD19+ IgD− CD27+ CD20− IgM− CD38+; CD38+/CD138+ B cells, CD19+ IgD− CD27+ CD38+ CD138+; Transitional B-cells, CD19+ CD24+ CD38+; Follicular B cells CD19+ IgD+ CD27− CD20+ CD24+ IgM^low^.

Flow cytometry fluorophor compensation for antibodies was performed using AbC™ Anti-Mouse Bead Kit (Life Technologies, Invitrogen, Foster City, CA, USA). Counting beads were added to each sample prior flow-cytometry analysis (AccuCheck Counting Beads, Life Technologies, Invitrogen, Foster City, CA, USA).

### Detection and characterization of YFV-specific CD8+ T cells

PBMCs and splenocytes were isolated and purified as described above. 2–4 × 10^6^ PBMCs or splenocytes were then incubated for 30 min at room temperature with a purified recombinant Fc protein (Human BD Fc block^TM^; BD Biosciences San Jose, CA, USA; 1/10 dilution) in order to prevent tetramer non-specific binding to Fc receptor. Cells were then incubated for 1 h at room temperature with a HLA-A*02:01 APC-conjugated tetramer (MBL International, Woburn, MA, USA; 1/10 dilution) specific for the NS4B 214–222 derived epitope (LLWNGPMAV)^44^. Cells were then incubated with the appropriate antibody cocktail optimized for CD8+ T-cell phenotyping and fixed as described above. Flow cytometry fluorophore compensation were performed as described above. Counting beads were added to each sample prior flow-cytometry analysis (AccuCheck Counting Beads, Life Technologies, Invitrogen, Foster City, CA, USA) and were used to determine the absolute number of YFV-specific CD8+ T cell (also referred NS4B/A2+ CD8+ T cells) per 100 µl of blood. Absolute count of splenic YFV-17D-specific CD8+ T cells were determined by reporting the number of total splenocytes and YFV-17D-specific CD8+ T cells processed by flow cytometry with the total count of splenocytes determined following tissue digestion. Number of events collected per mouse and for a given tissue to determine the absolute count of YFV-17D-specific CD8+ T cells ranged from 5 to 10 and from 100 to 700, in the blood and spleen, respectively. YFV-17D-specific CD8+ T cells were analyzed by flow-cytometry for the expression of HLA-DR, CD38, and Ki-67.

### T cell depletion experiment

NFA2-HIS/Flt3LG were injected with 100 µg of anti-CD4 or anti-CD8α (clone OKT-4 and OKT-8 respectively, BioXCell, West Lebanon, NH, USA) or not. Injections of 100 µg of antibody per mouse (intraperitoneal route) were performed for three consecutive days prior YFV-17D infection and three days post Adv-Flt3LG injection. A fourth antibody injection was performed 5 days post YFV-17D infection. At the day of infection, effective T-cell depletion was verified in the blood of animals by flow cytometry using antibodies targeting the following marker: human CD45+, mouse CD45+, human CD3+, human CD4+, and human CD8+. Weight and survival of the animals were monitored over a 20-day course of infection. Animals who survived the course of infection were sacrificed at day 20 post infection, and serum was collected to determine presence or absence of viral infection clearance in periphery. At the time of sacrifice, T cell count was determined in the blood and spleen of animals to control T cell depletion in mice treated with anti-CD4 or anti-CD8α. Additionally, the absolute counts of several myeloid cell populations (human cDCs, pDCs, monocytes, macrophages) and NK cells in the spleen were determined to ensure the specificity of the T-cell depletion.

### Cytokine quantification

Cytokine quantification was realized using the LEGENDplex^TM^ multi-analyte flow assay kit (Biolegend, San Diego, CA, USA). Sera from humanized mice were incubated for 2 h at room temperature with customized pre-mixed beads and detection antibodies specific for a panel of 13 human cytokines (IL-23, IL-12, IFNγ, TNFα, MCP-1, IL1β, IP-10, IL6, IFNβ, GM-CSF, IFNα2, IL18, IL33). Samples were then incubated for 30 min with SA-PE (Biolegend), wash and resulting fluorescent signals were analyzed on a flow cytometer (LSRII, BD Biosciences) according to manufacturer’s instructions. Analyte concentration was determined using LEGENDplex^TM^ software (Biolegend, San Diego, CA, USA).

### Bioluminescence imaging

NRG-HIS or NRGF-HIS were injected with 10^11^ AdV-Fluc particles. Ten days after injection, mice were anesthetized with isoflurane and injected intraperitoneally with 1.5 mg luciferin (Caliper Lifesciences, Waltham, MA, USA). Bioluminescence was quantified using an IVIS Lumina II platform (Caliper Lifesciences, Waltham, MA, USA).

### Isolation of human CD45+ and RNA extraction

To isolate human CD45+ cells, humanized mice were bled prior and following infection and total blood were collected. Cells were centrifuged and lysed with 1× lysis buffer (BD Pharm Lyse, BD Biosciences, San Jose, CA, USA) for 10 min at room temperature in the dark. Cells were then washed, counted, and resuspended at a concentration of 1 × 10^8^ cells/ml in a 2% (v/v) FBS–1 mM EDTA-PBS solution. Human CD45+ cells were then isolated using a human CD45+ cells enrichment kit (Stem Cell Technologies, Vancouver, British Columbia, Canada) according to the manufacturer’s protocol. Purification of human CD45+ cells was assessed by quantifying by flow cytometry human and murine CD45+ frequencies prior and after purification. Following enrichment, human CD45+ were spined and resuspended in RLT lysis buffer (Qiagen, Hilden, Germany). Cellular RNA was then extracted using the RNeasy Mini Kit (Qiagen, Hilden, Germany) following manufacturer’s instructions. For the transcriptomic experiments, mice were assembled as cohorts of 4–5 animals displaying humanization levels ranging between 50 and 80%. Following lysis and prior to cell resuspension in a 2% FBS–1 mM EDTA-PBS solution, blood samples of members of each cohort were pooled together, leading to one sample per cohort and time point (day 0 and day 11). Cohorts were assembled so the average humanization across cohorts differed only by ±5% between cohorts.

### Gene expression quantification by one-step RT-qPCR

Following cellular RNA isolation from human CD45+ cells (as described above), human MDA-5, STAT1, MX1, IRF7 and RSAD2 expression were quantified by one-step RT-qPCR using an iTaq Universal SYBR One-step kit (BioRad, Hercules, CA, USA). Expression was then normalized to the expression of human GAPDH. Primer sequences are as follows: MDA-5 sense, ACCAAATACAGGAGCCATGC; MDA-5 antisense, ACACGTTCTTTGCGATTTCC; STAT1 sense, TGGGTTTGACAAGGTTCTT; STAT1 antisense, TATGCAGTGCCACGGAAAG; MX1 sense, GTTTCCGAAGTGGACATCGCA; MX1 antisense, CTGCACAGGTTGTTCTCAGC; IRF7 sense, CCCACGCTATACCATCTACCT; IRF7 antisense, GATGTCGTCATAGAGGCTGTTG; RSAD2 sense, TGGGTGCTTACACCTGCTG; RSAD2 antisense, GAAGTGATAGTTGACGCTGGTT; GAPDH sense, GAAGGTGAAGGTCGGAGTC; GAPDH antisense, GAAGATGGTGATGGGATTTC.

### YFV-17D neutralization assay using mouse serum

Sera isolated from NFA2-NRGF-HIS/Flt3LG mice and identified by ELISA as containing YFV-specific antibodies following YFV-17D infection were employed for a neutralization assay. For each mouse, sera isolated prior (day 0) and after infection (day 40) were diluted at 1:20 and 1:40 in media containing YFV-17D viral particles (produced as described above). The number of viral particles per condition was determined as to reach a final m.o.i of 0.004 (200 p.f.u.). Viral particles and serum mixtures were incubated for 1 h at room temperature, and mixtures were used to infect naive Huh7.5 cells. As controls, we infected cells with cell culture media containing YFV-17D particles but no serum (also pre-incubated for 1 h at room temperature), or with media containing no viral particles and no serum. At 6 h post infection, media were replaced by a fresh methocell solution (DMEM, 10% (v/v) FBS, 1% (w/v) Methylcellulose). Four days post infection, cells were washed with PBS, fixed with 100% (v/v) ethanol for 25 min and stained with 0.1% (w/v) crystal violet. The percentage of infection inhibition was determined by counting and comparing the number of infectious plaques between the pre-infection serum and the post-infection serum condition, for each mouse and each given dilution.

### cDNA library generation and RNA-sequencing

The poly-A-containing RNA transcripts in the humanized mice total RNA samples were converted to cDNA and amplified following the Smart-seq2 method^57^. Sequencing libraries were made from the amplified cDNA samples using the Nextera kit (Illumina, San Diego, CA, USA), assigning a unique barcode to each of the libraries to be sequenced together. The cDNA samples and libraries were examined on the Bioanalyzer (Agilent Technologies, Santa Clara, CA, USA) DNA HS chips for size distribution, and quantified by Qubit fluorometer (Life Technologies, Invitrogen, Carlsbad, CA, USA). The majority of the cDNA fragments in each sample were between 1 and 3 kb, indicating good transcript integrity. The RNA-seq libraries from each experiment (i.e., one for each mouse strain, NRG-HIS and NRGF-HIS mice) were pooled together in equal amounts and sequenced on the Illumina HiSeq 2500 Rapid Flowcell as single-end 75nt reads following the standard protocol. Sequencing depth was of 10–30 million reads per sample (7–10 samples were loaded on a single flow cell). Raw sequencing reads were filtered by Illumina HiSeq Control Software and only Pass-Filter (PF) reads were used for further analysis.

### RNA-Sequencing data analysis

In the Galaxy platform^58^ through Princeton University, single-end short reads were mapped to the human reference (version GRCh38) and the murine reference (version GRCm38) using TopHat and Bowtie 2 with default parameters and read groups specified. Only counts from uniquely mapped reads were used in the DESeq 2 analyses.

Counts were generated by htseq-count^59^ version 0.6.1galaxy1 run on a Galaxy installation, downloaded, and read into R^60^ version 3.3.1 (2016-06-21) using scripts run in RStudio version 0.99.489^61^. The htseq count data was loaded into R and the DESeq2 (Galaxy Version 2.11.38) utilized^62^, capturing factors for cohort, day, and batch, with the sampling day as the experimental design. We set up the experimental design for the contrast we were interested in (day 0 versus day 11) and normalized log_2_ transformed transcript counts within DESeq2. Differential gene expression were determined for each group of mice (NRG-HIS, NRGF-HIS/Flt3LG or NRGF-HIS/Fluc) using the standard DESeq2 filters and nbinomWaldTest. Results were extracted from the DESeq2 analysis and annotated using Bioconductor’s AnnotationDbi (version 1.32.3) and org.Hs.eg.db packages (version 3.2.3). Differential gene expression was considered as significant when *p*_adj_ ≤ 0.05. Results are available in Supplementary Data 1.

Gene set enrichment analysis was performed in R using the package Generally Applicable Gene-set Enrichment (GAGE)^63^. Briefly, the GAGE method was called with the regularized log_2_ fold change values, and with the go.sets.hs^37,38^ or kegg.sets.hs^40,41^ database. Same.dir was set to true (since we were interested in whole-sale up- or down-regulated shifts). The top five resulting KEGG pathways (*q*-value ≤ 0.06) were then identified.

### Human vaccinees datasets and measure of correlation index

For the Emory cohort (*n* = 25 vaccinees, two independent trials of respectively 15 and 10 human vaccinees^28^), micro array data were downloaded from Gene Expression Omnibus under series accession no. GSE13485. For the Lausanne and Montreal cohorts (*n* = 11 and *n* = 25 vaccinees respectively^44^), data were downloaded from Gene Expression Omnibus under series accession no. GSE13699. For the three cohorts, micro array raw data at day 0 and day 7 post vaccination were downloaded and we employed GEO2R (https://www.ncbi.nlm.nih.gov/geo/geo2r/) to determine the set of differentially regulated genes at day 7 post vaccination. For the Emory cohort, we identified a set of 193 genes with *p*_adj_ ≤ 0.1 differentially regulated at day 7 post vaccination. For the Lausanne and Montreal cohort, we identified a set of 2674 and 12930 genes with *p*_adj_ ≤ 0.1 differentially regulated at day 7 post vaccination, respectively.

To establish our correlation index, we decided to focus on gene expression fold-change (*FC*) as the compared variable, as it is adimensional and can therefore be compared across experiments using different expression measurement methodologies

We first sought to establish a global human dataset of genes that could be used to measure similarity with humanized mice in a relevant fashion. To do so, we used two methods. In the first one (unbiased), the gene set included all genes that had been statistically determined to be differentially expressed (for a defined Benjamini–Holmes-adjusted *p*-value threshold of *q* equal or lower than 0.1) in at least one of the three human cohorts. This method has the merit of being the most unbiased, as it uses no other criteria than statistical relevance. However, it is sensitive to false positives (that can occur somewhat frequently in transcriptome analyses, especially concerning low-expression genes). Moreover, when constructing the gene dataset that way, a disproportionate fraction of it was actually determined by the Montreal human cohort, as this cohort displayed the highest number of differentially regulated genes. To compensate for the over-weight of the Montreal cohort in our dataset, we therefore also used a double selection method, where we only selected genes that had been statistically determined to be differentially expressed (for a defined Benjamini–Holmes-adjusted *p*-value threshold of *q* equal or lower than 0.1) in two or more of the three human cohorts. This method ensures the biological relevance of the genes selected in the set construction as they have been detected in more than one cohort, but at the cost of omitting potentially relevant candidates that were only detected in only one cohort.

For both methods, after elimination of duplicates, we averaged the fold-changes of the genes in the set across all three human cohorts, therefore building two adjusted-*p*-value-dependent reference vectors: the “unbiased” vector *U*_*q*_ and the “double selection” vector *D*_*q*_.

Finally, the Spearman rho correlation statistics to those reference vectors were computed for each humanized mouse cohort to build our correlation index. For a mouse cohort *M*, the “unbiased” value vector *M*_*U,q*_ was first built as follows:

$$M_{U,q} = \left\{ {FC_g,g\,{\mathrm{in}}\,{\mathrm{unbiased}}\,{\mathrm{gene}}\,{\mathrm{set}}} \right\}$$

The values in *M*_*U,q*_ and *U*_*q*_ were ranked to obtain the vectors rg*M*_*U,q*_ and rg*U*_*q*_, and the

unbiased correlation index *r*_*M,U,q*_ (simply referred to as *r*_*U*_ in the Results section) was then computed as follows:

$$r_{M,U,q} = {\mathrm{cov}}({\mathrm{rg}}M_{U,q}{\mathrm{rg}}U_q)/(\sigma _{{\mathrm{rg}}}M_{U,q}\sigma _{{\mathrm{rg}}}U_q)$$

The double selection correlation index *r*_*M,D,q*_ (simply referred to as *r*_*D*_ in the Results section) was computed in a similar fashion for a mouse cohort *M*.

It is important to note that the correlation index is dependent on the adjusted *p*-value threshold selected when building the reference vectors *U*_*q*_ and *D*_*q*_. As this threshold is raised, more genes will be included in building the index, thus affecting the whole process leading to the computing of our correlation indexes. To address that question, we built several versions of our reference vectors *U*_*q*_ and *D*_*q*_ using different adjusted *p*-value thresholds (from 0.01 to 0.1 by 0.01 increments) and computed *r*_*M,U,q*_ and *r*_*M,D,q*_ for each of them. Based on the shapes of the *r*_*M,U,q*_ = f(q) and *r*_*M,D,q*_ = f(q) curves, which all show a steady, linear decline with no sharp discontinuity between q = 0.02 and *q* = 0.08, we chose to use the correlation index value derived from gene sets constructed with an adjusted *p*-value threshold of 0.05 as our definitive correlation indexes. For a mouse cohort M, the unbiased correlation index to human cohorts *r*_*M,U*_ is therefore:

$$r_{M,U} = r_{M,U,q},q = 0.05({\mathrm{simply}}\,{\mathrm{referred}}\,{\mathrm{to}}\,{\mathrm{as}}\,r_{U,q = 0.05}{\mathrm{in}}\,{\mathrm{the}}\,{\mathrm{Results}}\,{\mathrm{section}})$$

And the double selection index *r*_*M,D*_:

$$r_{M,D} = r_{M,D,q},q = 0.05({\mathrm{simply}}\,{\mathrm{referred}}\,{\mathrm{to}}\,{\mathrm{as}}\,r_{D,q = 0.05}{\mathrm{in}}\,{\mathrm{the}}\,{\mathrm{Results}}\,{\mathrm{section}})$$

### Protein–protein interaction network representation

Network coordinates and protein–protein interactions were downloaded via the STRING biological database^64^ and rendered using the open source software platform for network data integration Cytoscape V3.4.0^65^.

### scRNA-Seq

Human CD45+ and human CD33+ populations from the spleens of NFA2-HIS/Flt3LG mice and NRG-HIS mice were FACS-sorted and loaded onto prepared Seq-Well arrays, as previously described. Briefly, 10,000 single cells were loaded onto one array containing 86,000 barcoded mRNA capture beads. Cells and beads were co-confined in microwells on the array, and a polycarbonate membrane sealed individual wells to allow for isolated single-cell lysis and transcript hybridization prior to bead recovery for reverse transcription. Next, each library was treated with Exonuclease I to remove excess primers, and PCR amplification was performed using KAPA Hifi PCR Mastermix (Thermo Scientific, Waltham, MA, USA) to generate final cDNA libraries. Libraries were then constructed using the Nextera XT DNA tagmentation method (Illumina, San Diego, CA, USA). Tagmented and amplified libraries were subsequently purified and sequenced using an Illumina 75 cycle NextSeq500/550v2 kit (read 1: 20 12 bp barcode, 8 bp UMI; read 2: 50). Detailed procedures for scRNA-Seq data alignment and analysis are available in the Methods section.

### ScRNA-sequencing library generation

Frozen splenocytes from NFA2-HIS/Flt3LG and NRG-HIS mice were thawed and stained with antibodies for anti-human CD33 or CD45, and calcein green as a positive viability marker. Cell populations that were either anti-human CD33+ or anti-human CD45+ and calcein green positive were sorted into RPMI + 10% FBS. Single cells from each sort gate and each animal were loaded onto prepared Seq-Well arrays, as previously described^49^. Briefly, 10,000 single cells from each sort gate were loaded onto one array containing 86,000 barcoded mRNA capture beads. Cells and beads were co-confined in microwells on the array, and a polycarbonate membrane sealed individual wells to allow for isolated single-cell lysis and transcript hybridization prior to bead recovery for reverse transcription. Next, each library was treated with Exonuclease I to remove excess primers, and PCR amplification with KAPA Hifi PCR Mastermix (Thermo Scientific, Waltham, MA, USA) to generate final cDNA libraries. Libraries were then constructed using the Nextera XT DNA tagmentation method (Illumina, San Diego, CA, USA). Tagmented and amplified libraries were subsequently purified and sequenced using an Illumina 75 cycle NextSeq500/550v2 kit (30 bp PE reads).

### ScRNA-Seq alignment and analysis

The reads were aligned as described in ref. ^66^. Briefly, for each NextSeq sequencing run, raw sequencing data was converted to demultiplexed FASTQ files using bcl2fastq2 based on Nextera N700 indices corresponding to individual samples/arrays. Reads were then aligned simultaneously to both mm10 and hg19 genomes using the Galaxy portal maintained by the Broad Institute for Drop-Seq alignment using standard settings of the STAR aligner. Individual reads were tagged according to the 12-bp barcode sequenced and the 8-bp UMI contained in Read 1 of each fragment. Following alignment, reads were binned onto 12-bp cell barcodes and collapsed by their 8-bp UMI. Digital gene expression matrices for each sample were obtained from quality filtered and mapped reads. UMI-collapsed data was utilized as input into R for further analysis.

We first compared the alignment quality between NFA2-HIS/Flt3LG mice and NRG-HIS mice in each sorting condition. We confirmed equivalent sequencing depth and sample input quality between NFA2-HIS/Flt3LG and NRG-HIS mice by observing the total transcripts/single cell detected for the mouse single cells that contaminated our sorted populations. However, we observed overall lower quality among single cells that align to human genomes in the NRG-HIS mice compared to NFA2-HIS/Flt3LG mice, which cannot be attributed to differences in sequencing depth. We next merged UMI matrices across all genes detected in any hCD45+ sample (2 from NFA2-HIS/Flt3LG, 2 from NRG-HIS mice), and eliminated cells with fewer than 300 UMI detected and fewer than 300 unique genes detected (*n* = 1297 across 2 NFA2-HIS/Flt3LG mice, *n* = 457 across 2 NRG-HIS mice). We next partitioned the matrix into cells originating from NFA2-HIS/Flt3LG mice or NRG-HIS mice, and completed all clustering and cell calling analyses in parallel. To complete dimensionality reduction and data visualization methods, we first identified the top variable genes by including all genes with an average normalized and scaled expression value greater than 0.32 and dispersion greater than 0.6. Principal Components Analysis was performed over the list of variable genes and all cells (for each experimental group individually). We determined the top significant principal components using a permutation method as previously described^67^. Significant principal components were used for tSNE plotting, with perplexity of 40. We used FindClusters, a clustering algorithm in the R package Seurat 1.4.0.1 (https://github.com/satijalab/seurat) to identify significant clusters. 10 clusters were found for NFA2-HIS/Flt3LG mice, and 6 clusters were found for NRG-HIS mice, using equivalent parameters. No cluster was attributed to any single mouse. To identify genes that defined each cluster, we performed a likelihood ratio test implemented in Seurat. Top marker genes were used to classify cell clusters into cell types based on existing biological knowledge.

To better understand diversity within the non-T cell, non-B cell compartment of each humanized mouse type, we merged both hCD45+ and hCD33+ arrays for NFA2-HIS/Flt3LG mice, and separately merged hCD45+ and hCD33+ arrays from NRG-HIS mice. We eliminated low quality cells with the same parameters as above, and eliminated any cell with expression of the following genes: CD3E, CD3G, CD3D, TRAC*, TRBC*, CD79A, CD79B, IGKC, IGLC*, MSA41, CD19 (* indicates any number), to gate out any T or B cells. We applied the same protocol as above to identify variable genes, identify significant principal components, calculate a tSNE representation, find significant cell clusters, and annotate cell clusters by identifying biologically meaningful defining genes. Genes that define each of these non-T, non-B cell clusters in the NFA2-HIS/Flt3LG mice were compared directly to the expression of these genes in the NRG-HIS non T, non B cells. Gene differential expression was calculated using a likelihood ratio test for zero inflated data, implemented in Seurat, with original description described^68^.

To directly compare the abundance of each cell type by annotation in each humanized mouse dataset, we took all hCD45+ samples and merged their UMI matrices. We analyzed this data as described above, and calculated the frequency of each cell type within either NRG-HIS mice or NFA2-HIS/Flt3LG mice.

### Statistical analysis

Statistical analyses were performed using either a non-parametric Wilcoxon–Mann–Whitney test^69^ or a parametric Student’ *t* test (GraphPad Prism software V6.0) when appropriate and as indicated in each figure legend. A two-way ANOVA test was performed for multiple comparison (GraphPad Prism software V6.0). *p ≤ 0.05, **p ≤ 0.01, ***p ≤ 0.001, ****p ≤ 0.0001. Information related to the statistical analysis performed for RNA-seq and single-cell RNA seq experiments can be found in the related sections above.

## Electronic supplementary material

Descriptions of Additional Supplementary Files

Supplementary Information

Supplementary Data 1

Supplementary Data 2

Supplementary Data 3

## Data Availability

All the transcriptomic datasets generated for this study are available through the National Center for Biotechnology Information Gene Expression Omnibus (GEO) under the SuperSeries accession no. GSE119751. The bulk RNA-seq data and scRNA-seq data are referenced under two SubSeries that are linked to GSE119751, respectively GSE119749 and GSE119750. All other relevant data, as well as additional information regarding reagents employed in this study, are available from the authors upon request.
