# Supplementary material for: Selective expansion of myeloid and NK cells in humanized mice yields human-like vaccine responses
Source: Nat Commun. 2018 Nov 28;9:5031. doi: 10.1038/s41467-018-07478-2 (PMC6262001; doi:10.1038/s41467-018-07478-2)
Supplement: Supplementary file 1 — Descriptions of Additional Supplementary Files [file 41467_2018_7478_MOESM1_ESM.docx]

**Description of Additional Supplementary Files**

File Name: **Supplementary Data 1**

Description: Excel file containing the list of differentially expressed genes (*p_adj_*≤0.1) in the peripheral blood mononuclear cells of different humanized mouse models (NRG-HIS, NRGF-HIS/Fluc and NRGF-HIS/Flt3LG) upon YFV-17D infection.

File Name: **Supplementary Data 2**

Description: Excel file containing the scRNA-Seq cluster annotations used for generating the **Figure 8** and **Figure 9**.

File Name: **Supplementary Data 3**

Description: Excel file containing the scRNA-Seq differential expression results and gene ontology analysis of NK cells and macrophages, used for generating the **Supplementary Figure 12**.
