# Supplementary material for: Selective expansion of myeloid and NK cells in humanized mice yields human-like vaccine responses
Source: Nat Commun. 2018 Nov 28;9:5031. doi: 10.1038/s41467-018-07478-2 (PMC6262001; doi:10.1038/s41467-018-07478-2)
Supplement: Supplementary file 2 — Supplementary Information [file 41467_2018_7478_MOESM2_ESM.pdf]

SUPPLEMENTARY INFORMATION

FOR

Selective expansion of myeloid and NK cells in humanized mice yields human-like vaccine responses

Douam et al.

CONTENT:

1. Supplementary Figures

Supplementary Figure 1. Immune response to YFV-17D in NRG-HIS mice.
Supplementary Figure 2. Characterization of the NRGF-HIS mouse model.
Supplementary Figure 3. Gating strategy for quantifying human and mouse immune cell subsets in humanized mice.
Supplementary Figure 4. Transcriptomic signatures in NRGF-HIS/Fluc and Flt3LG mice upon YFV-17D infection.
Supplementary Figure 5. Gene ontology (GO) enrichment analysis of the transcriptomic signatures of infected NRG-HIS and NRGF-HIS mice.
Supplementary Figure 6. Clinical symptoms and cytokine profiling in infected NRG-HIS/Flt3LG and NRGF-HIS/Flt3LG mice.
Supplementary Figure 7. Immune cell subset frequencies in YFV-17D infected NRG-HIS/Flt3LG and NRGF-HIS/Flt3LG mice.
Supplementary Figure 8. Antigen-specific cellular responses upon YFV-17D infection in NFA2-HIS/Fluc and NFA2-HIS/Flt3LG mice.
Supplementary Figure 9. T cell function in NFA2-HIS/Fluc and NFA2-HIS/Flt3LG mice during YFV-17D infection.
Supplementary Figure 10. B-cell differentiation in NFA2-HIS/Fluc and NFA2-HIS/Flt3LG mice during YFV-17D infection.
Supplementary Figure 11. scRNA-Seq profiling of NRG-HIS and NFA2-HIS/Flt3LG splenocytes: Data quality and cellular abundances.
Supplementary Figure 12: Candidate ILCs and functional differences among splenic myeloid populations in NRG-HIS and NFA2-HIS/Flt3LG mice.

2. **Supplementary Tables**

Supplementary Table 1. Transcriptomic correlation index of humanized mouse models with human vaccinees.

3. Supplementary Notes

Supplementary Note 1. Analysis of the cellular complexity of the immune system in humanized mice by single-cell RNA sequencing analysis.

SUPPLEMENTARY FIGURES

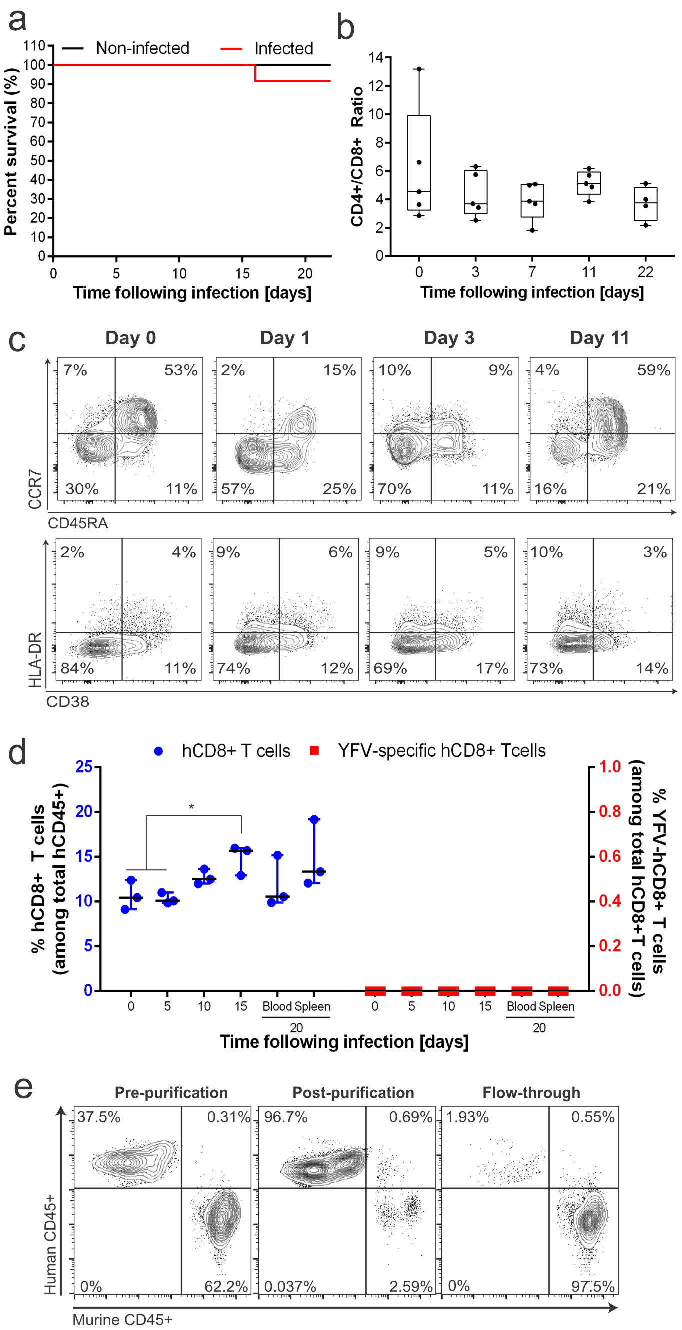

Supplementary Figure 1. Immune response to YFV-17D in NRG-HIS mice.
a. Percentage of survival of NRG-HIS mice over a course of 22 days after YFV-17D infection (red line, n=5). A cohort of five non-infected NRG-HIS mice were monitored in parallel (black line). **b.** Ratio between frequencies of peripheral human CD4+ and CD8+ T cells in the blood of NRG-HIS mice over the course of YFV-17D infection. Bounds of box and whiskers represent the min-to-max CD4+/CD8+ ratio at each time point. Medians are indicated in each box as center line (n=5). **c.** Representative flowcytometric plots of CCR7 vs. CD45RA (top) and HLA-DR+ vs. CD38+

(bottom) cells among human CD3+CD8+ T cells isolated from the spleen of NRG-HIS mice (n=3-6) following YFV-17D infection. Frequencies are reported for different times pre- and post-infection (days 0, 1, 3 and 11 post infection). **d.** Percentage of peripheral human CD8+ T cells (blue) and YFV-specific CD8+ T cells (red) in the blood of NRG-HIS-HLA-A2 mice over the course of YFV-17D infection among the total human CD45+ and the total human CD8+ T cell populations, respectively. Percentage of human CD8+ T cells and YFV-specific CD8+ T cells in the spleen at time of sacrifice (day 20 post infection) are also shown. *$p \leq 0.05$ (Student's t test). For each time point, median (black horizontal line) with range (min-to-max frequencies) is shown (n=3). **e.** Purification of human CD45+ cells from the peripheral blood of humanized mice. Representative FACS dot plots displaying human CD45+ and murine CD45+ frequencies before and after purification and in the flow-through.

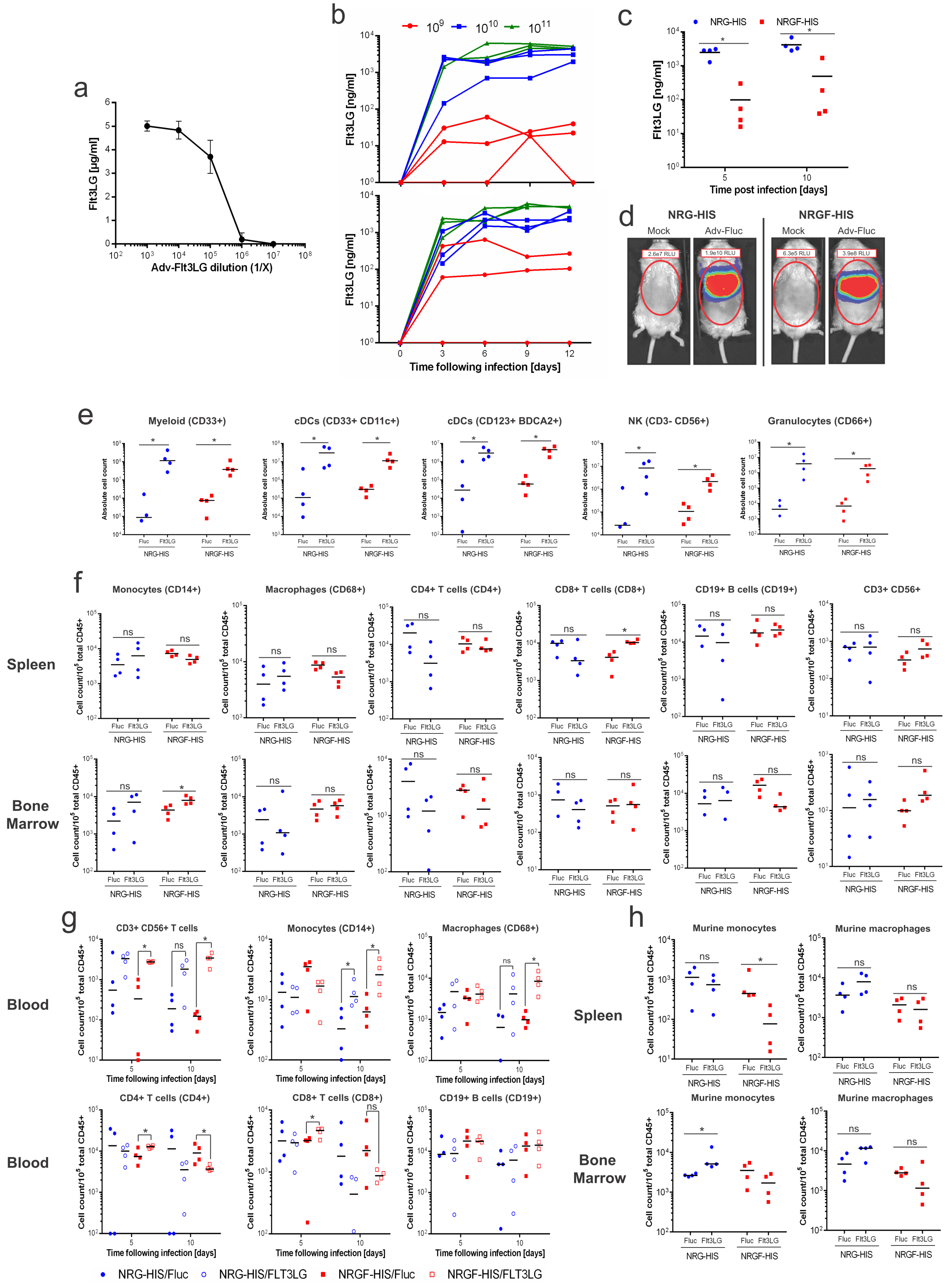
a
Flt3LG [µg/ml]
Adv-Flt3LG dilution (1/X)
b
Flt3LG [ng/ml]
Time following infection [days]
c
NRG-HIS
NRGF-HIS
Flt3LG [ng/ml]
Time post infection [days]
d
NRG-HIS
NRGF-HIS
Mock
Adv-Fluc
e
Myeloid (CD33+)
cDCs (CD33+ CD11c+)
cDCs (CD123+ BDCA2+)
NK (CD3- CD56+)
Granulocytes (CD66+)
Absolute cell count
f
Spleen
Bone Marrow
Monocytes (CD14+)
Macrophages (CD68+)
CD4+ T cells (CD4+)
CD8+ T cells (CD8+)
CD19+ B cells (CD19+)
CD3+ CD56+
Cell count/10^5 total CD45+
g
Blood
CD3+ CD56+ T cells
Monocytes (CD14+)
Macrophages (CD68+)
CD4+ T cells (CD4+)
CD8+ T cells (CD8+)
CD19+ B cells (CD19+)
Time following infection [days]
h
Spleen
Bone Marrow
Murine monocytes
Murine macrophages
NRG-HIS/Fluc
NRG-HIS/FLT3LG
NRGF-HIS/Fluc
NRGF-HIS/FLT3LG

Supplementary Figure 2. Immunological characterization of the NRGF-HIS mouse model. **a.** Concentration of human Flt3LG in cell culture supernatant following infection of HEK293T cells with AdV-Flt3LG. HEK293T were infected with different dilutions of Adv-Flt3LG (10^3 to 10^7 particles) and human Flt3LG concentration determined three days post infection (Mean±s.d.; n=3). **b.** Human Flt3LG concentration in the sera of non-engrafted NRG (top) and NRGF (bottom) mice following infection with AdV-Flt3LG (10^9, 10^{10} or 10^{11} particles) (n=3 per group and dose). **c.** Human Flt3LG concentration in the sera of NRG-HIS (blue) and NRGF-HIS (red) mice following infection with 10^{10} AdV-Flt3LG particles. Medians are shown as horizontal black lines (n=4 per group). **p*≤0.05 (Wilcoxon-Mann-Whitney test). **d.** Luciferase expression in NRG-HIS and NRGF-HIS mice infected with 10^{11} AdV-Fluc particles at day 10 post infection. Representative images of luciferase expression within each type of mouse, mock-infected or infected with AdV-Fluc. Relative light unit (RLU) for indicated area (red circle) is shown. **e**. Absolute count of several human splenic cell subsets in NRG-HIS (blue circle) and NRGF-HIS (red square) mice following injection with AdV-Fluc or Adv-Flt3LG. The same data are reported as frequencies of total CD45+ cells in **Fig.3c**. cDCs, conventional dendritic cells; pDCs, plasmacytoid dendritic cells; NK, natural killer cells. **f-g.** Expansion of human immune cell subsets in the spleen (**f**), bone marrow (**f**) or blood (**g**) of NRG-HIS (blue circle) and NRGF-HIS (red square) mice following injection with AdV-Fluc (closed circle/square in panel G) or Adv-Flt3LG (open circle/square in panel G). NKT, T lymphocyte natural killer cells. **h.** Expansion of murine monocytes and macrophages in the spleen and bone marrow of NRG-HIS and NRGF-HIS mice following injection with AdV-Flt3LG or AdV-Fluc. For panels **e-h**, medians are shown as horizontal black lines for each experimental condition (n=4 per group). **p*≤0.05, ns non-significant (Wilcoxon-Mann-Whitney test).

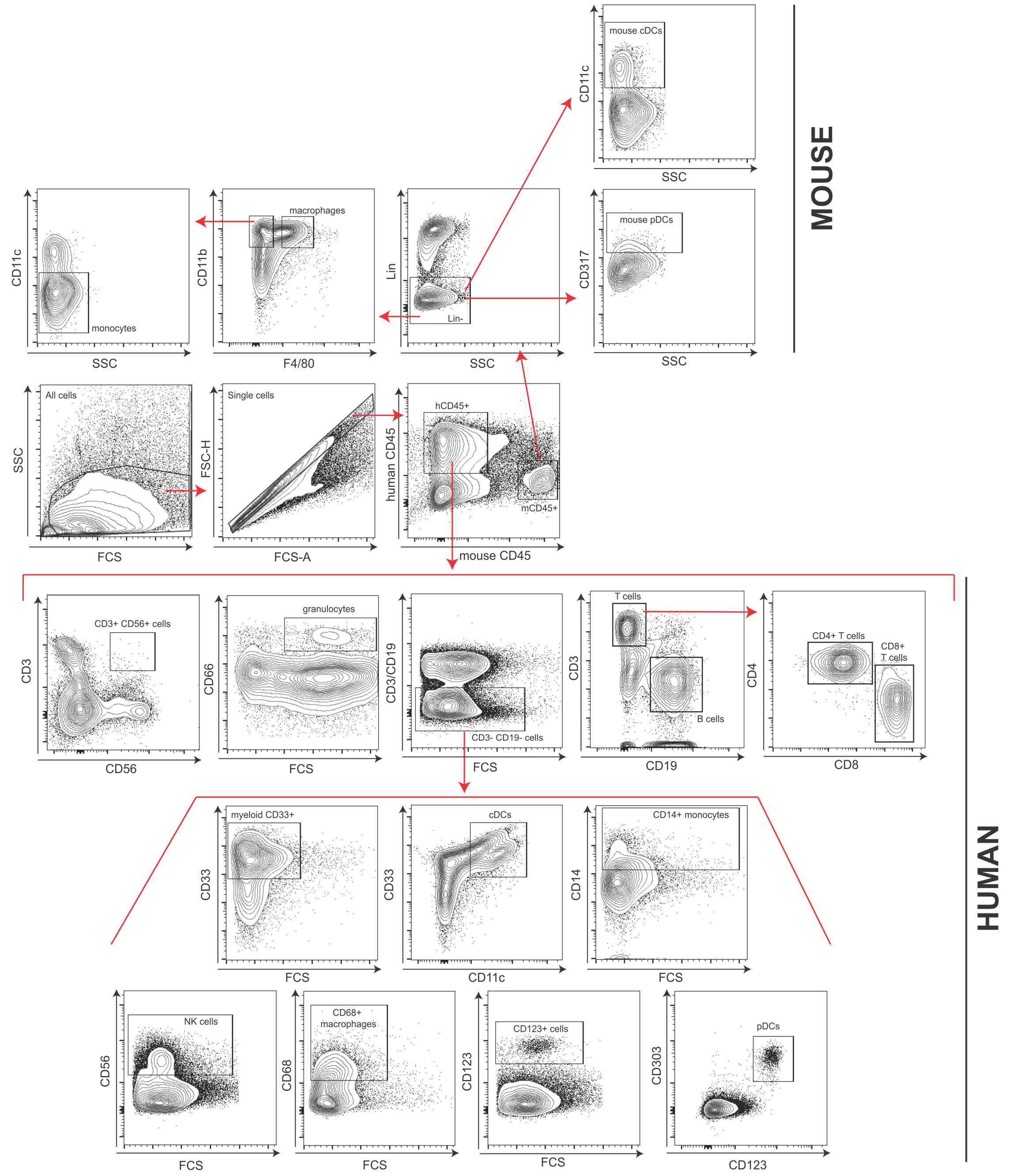

Supplementary Figure 3. Gating strategy for quantifying human and mouse immune cell subsets in humanized mice.

Gating strategy used to generate the data shown in **Fig.1b**, **Fig.3c-e**, **Supplementary Figure 1b**, **Supplementary Figure 2e-h**, **Supplementary Figure 7** and **Supplementary Figure 9b-d.** Red arrows indicate the direction of the gating strategy: their tails are located within the parent population gate and their heads point to the parental population gated into. cDCs, conventional dendritic cells; hCD45+, human CD45+ cells; mCD45+, mouse CD45+ cells; pDCs, plasmacytoid dendritic cells; Lin, CD3- CD19- NK1.1- TER119- Ly-6G/Gr1- cells; NK cells, natural killer cells.

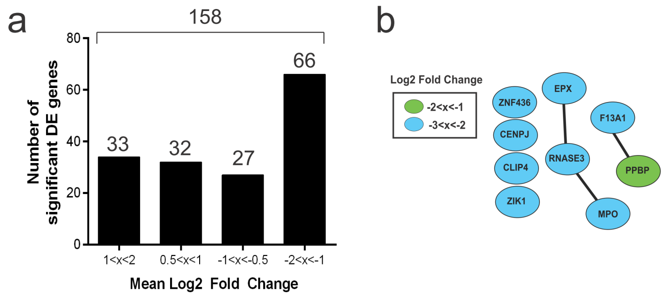

Supplementary Figure 4. Transcriptomic signatures in NRGF-HIS/Fluc and NRGF-HIS/Flt3LG mice upon YFV-17D infection.

a. Number of significantly differentially expressed genes identified across the three cohorts of NRGF-HIS/Flt3LG and grouped by mean log2 FC: 1<x<2, 0.5<x<1, -1<x<-0.5 and -1<x<-2 (out of a total of 158 genes). **b.** Protein-protein interaction network displaying the 9 genes DE upon YFV-17D infection of NRGF-HIS/Fluc mice. Each gene is colored based on its log_2FC (-2<x<-1, green; -3<x<-2, blue).

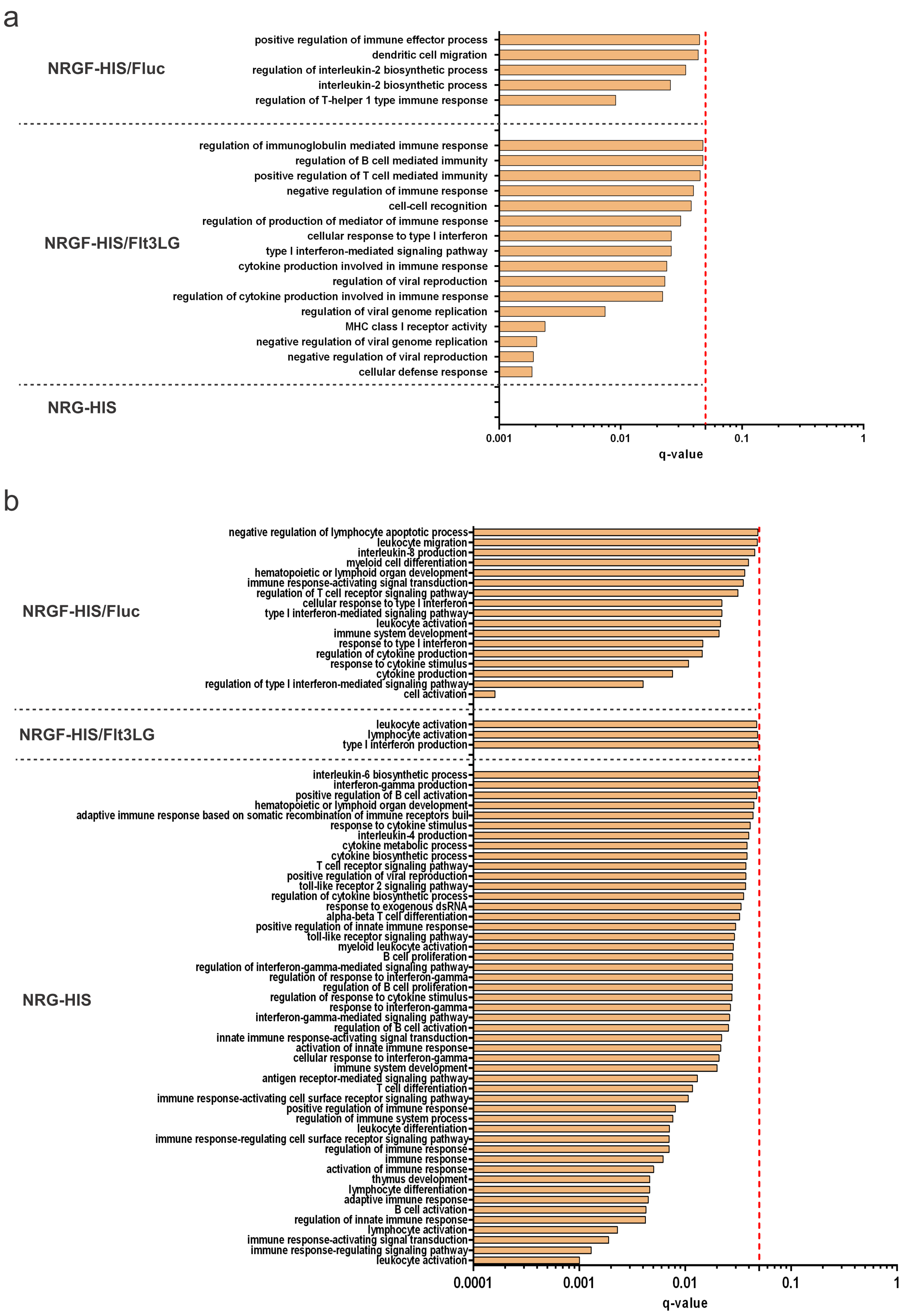
a
NRGF-HIS/Fluc
positive regulation of immune effector process
dendritic cell migration
regulation of interleukin-2 biosynthetic process
interleukin-2 biosynthetic process
regulation of T-helper 1 type immune response
NRGF-HIS/Flt3LG
regulation of immunoglobulin mediated immune response
regulation of B cell mediated immunity
positive regulation of T cell mediated immunity
negative regulation of immune response
cell-cell recognition
regulation of production of mediator of immune response
cellular response to type I interferon
type I interferon-mediated signaling pathway
cytokine production involved in immune response
regulation of viral reproduction
regulation of cytokine production involved in immune response
regulation of viral genome replication
MHC class I receptor activity
negative regulation of viral genome replication
negative regulation of viral reproduction
cellular defense response
NRG-HIS
0.001
0.01
0.1
1
q-value
b
NRGF-HIS/Fluc
negative regulation of lymphocyte apoptotic process
leukocyte migration
interleukin-8 production
myeloid cell differentiation
hematopoietic or lymphoid organ development
immune response-activating signal transduction
regulation of T cell receptor signaling pathway
cellular response to type I interferon
type I interferon-mediated signaling pathway
leukocyte activation
immune system development
response to type I interferon
regulation of cytokine production
response to cytokine stimulus
cytokine production
regulation of type I interferon-mediated signaling pathway
cell activation
NRGF-HIS/Flt3LG
leukocyte activation
lymphocyte activation
type I interferon production
NRG-HIS
interleukin-6 biosynthetic process
interferon-gamma production
positive regulation of B cell activation
hematopoietic or lymphoid organ development
adaptive immune response based on somatic recombination of immune receptors buil
response to cytokine stimulus
interleukin-4 production
cytokine metabolic process
cytokine biosynthetic process
T cell receptor signaling pathway
positive regulation of viral reproduction
toll-like receptor 2 signaling pathway
regulation of cytokine biosynthetic process
response to exogenous dsRNA
alpha-beta T cell differentiation
positive regulation of innate immune response
toll-like receptor signaling pathway
myeloid leukocyte activation
B cell proliferation
regulation of interferon-gamma-mediated signaling pathway
regulation of response to interferon-gamma
regulation of B cell proliferation
regulation of response to cytokine stimulus
response to interferon-gamma
interferon-gamma-mediated signaling pathway
regulation of B cell activation
innate immune response-activating signal transduction
activation of innate immune response
cellular response to interferon-gamma
immune system development
antigen receptor-mediated signaling pathway
T cell differentiation
immune response-activating cell surface receptor signaling pathway
positive regulation of immune response
regulation of immune system process
leukocyte differentiation
immune response-regulating cell surface receptor signaling pathway
regulation of immune response
immune response
activation of immune response
thymus development
lymphocyte differentiation
adaptive immune response
B cell activation
regulation of innate immune response
lymphocyte activation
immune response-activating signal transduction
immune response-regulating signaling pathway
leukocyte activation
0.0001
0.001
0.01
0.1
1
q-value

Supplementary Figure 5. Gene ontology (GO) enrichment analysis of the transcriptomic signatures of infected NRG-HIS and NRGF-HIS mice.

List of GO terms significantly (*q-value*≤0.05) upregulated (**a**) or downregulated (**b**) in PBMCs of NRG-HIS, NRGF-HIS/Flt3LG and NRGF-HIS/Fluc mice following FC replicate analysis. A red dotted line symbolizes a *q-value* of 0.05.

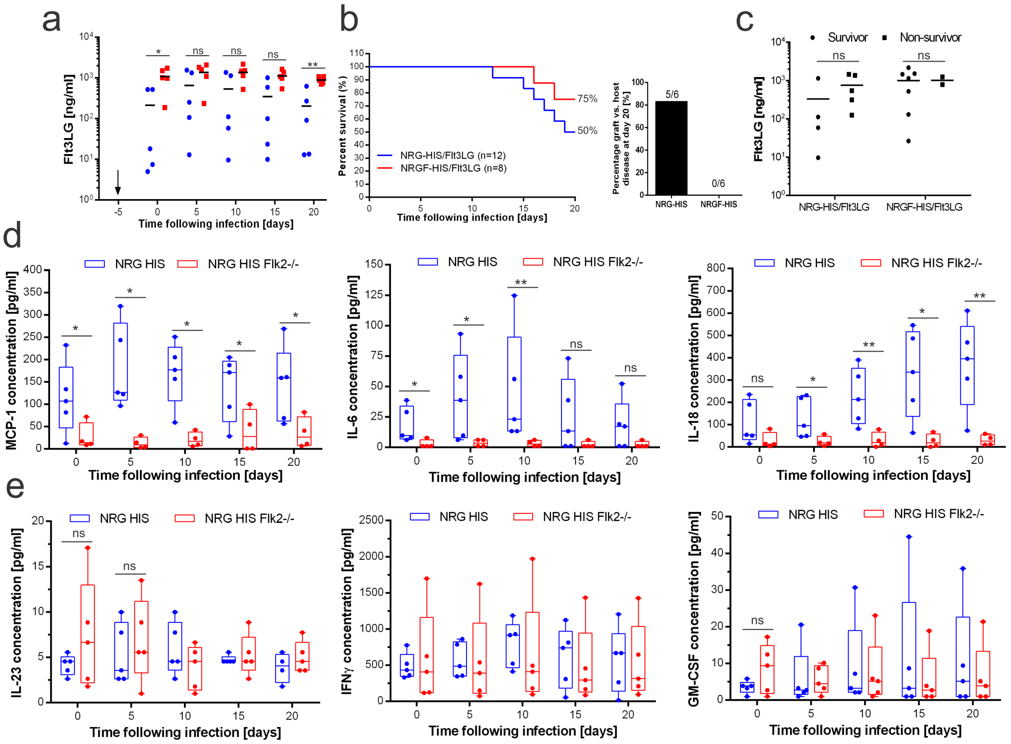

Supplementary Figure 6. Clinical symptoms and cytokine profiling in infected NRG-HIS/Flt3LG and NRGF-HIS/Flt3LG mice.

a. Human Flt3LG concentration in the sera of five infected NRG-HIS/Flt3LG (blue) and NRGF-HIS/Flt3LG (red) mice Median are shown as horizontal black lines (n=5 per group). *p≤0.05, **p≤0.01, ns non-significant (Wilcoxon-Mann-Whitney test). **b.** Percent survival of NRG-HIS/Flt3LG (blue, n=12) and NRGF-HIS/Flt3LG (red, n=8) mice over a course of 20 days after YFV-17D infection. The percentage of surviving NRG-HIS/Flt3LG and NRGF-HIS/Flt3LG mice displaying signs of host versus graft disease (GVHD) at day 20 post infection are shown in the right panel. **c**. Human Flt3LG concentration in the serum of infected NRG-HIS/Flt3LG (blue, n=9) and NRGF-HIS/Flt3LG (red, n=9) mice that survived (survivor, closed circle) or not (non-survivor, closed square) the 20-day course of infection. Concentrations were measured 15 days following AdV-Flt3LG injection. Medians are shown as horizontal black lines. ns non-significant (Wilcoxon-Mann-Whitney test). **d-e.** Cytokine/chemokine concentrations in the serum of NRG-HIS (blue) and NRGF-HIS (red) mice over the course of infection following YFV-17D infection. Concentration of seven major pro-inflammatory cytokines (MCP-1, IL-6, IL-18 and IP-10; **d**) and three additional cytokines (IL-23, IFNγ and GM-CSF; **e**) was determined by flow-cytometry beads array. Bounds of box and whiskers represent the min-to-max cytokine concentration at each time point. Medians are indicated in each box as center line (n=4-5 per group). *p≤0.05, **p≤0.01, ns non-significant (Wilcoxon-Mann-Whitney test).

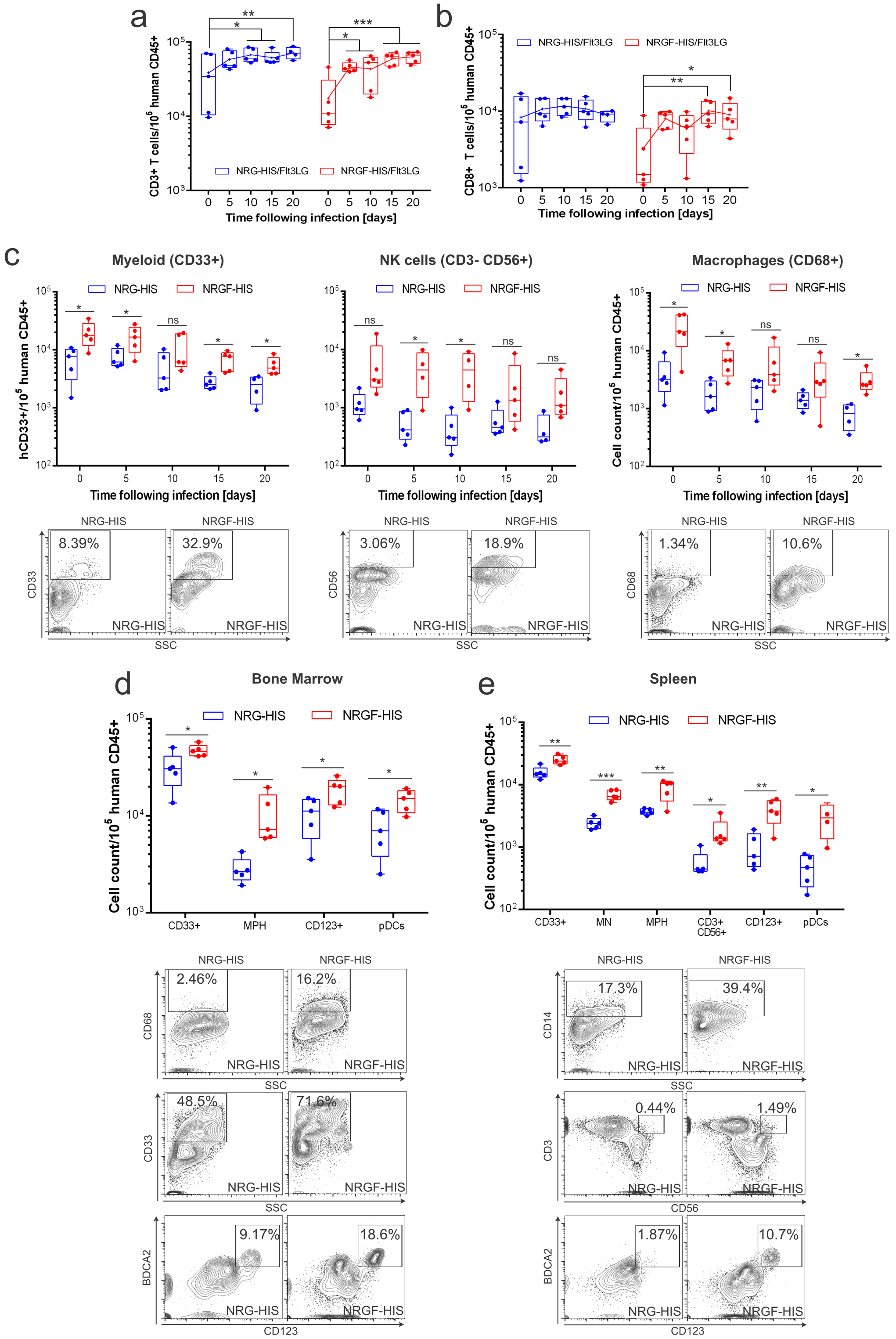
a
b
c
d
e
CD3+ T cells/10^5 human CD45+
CD8+ T cells/10^5 human CD45+
NRG-HIS/Flt3LG
NRGF-HIS/Flt3LG
Time following infection [days]
Myeloid (CD33+)
NK cells (CD3- CD56+)
Macrophages (CD68+)
NRG-HIS
NRGF-HIS
hCD33+/10^5 human CD45+
Cell count/10^5 human CD45+
8.39%
32.9%
3.06%
18.9%
1.34%
10.6%
SSC
Bone Marrow
Spleen
CD33+
MPH
CD123+
pDCs
MN
CD3+ CD56+
2.46%
16.2%
48.5%
71.6%
9.17%
18.6%
17.3%
39.4%
0.44%
1.49%
1.87%
10.7%
CD68
CD33
BDCA2
CD123
CD14
CD3
CD56

Supplementary Figure 7. Immune cell subset frequencies in YFV-17D infected NRG-HIS/Flt3LG and NRGF-HIS/Flt3LG mice.

a-b. Changes in the frequencies of CD3+ T cells (**a**) and CD3+ CD8+ T cells (**b**) in the blood of NRG-HIS (blue) and NRGF-HIS (red) mice over the course of YFV-17D infection. (n=5 per group). *p≤0.05, **p≤0.01, ***p≤0.001 (two-way ANOVA). **c**. Frequencies of peripheral human myeloid cells (left), NK cells (middle) and MPHs (right) in the blood of NRG-HIS (blue) and NRGF-HIS (red) mice over the course of infection. Flowcytometric plots displaying representative frequencies for each subset and type of mouse at a given time point (myeloid cells and macrophages, day 5 post infection; NK cells, day 10 post infection) are shown**.** Medians indicated in each box and whiskers (n=5 per group). *p≤0.05, ns, non-significant (Wilcoxon-Mann-Whitney test).. **d-e**. Proliferation of specific human immune cell subsets in the bone marrow (**d**) and spleen (**e**) of NRG-HIS (blue) and NRGF-HIS (red) mice at the time of sacrifice (day 20 post infection) (n=5 per group). Flowcytometric plots display representative frequencies of MPHs (CD68+), myeloid cells (CD33+) and pDCs (CD123+ BDCA2+) in the bone marrow **(d)** and of MNs (CD14+), CD3+ CD56+ T cells and pDCs (CD123+ BDCA2+) in the spleen **(e)**. *p≤0.05, **p≤0.01, ***p≤0.001 (Wilcoxon-Mann-Whitney test). CD3+ CD56+, CD3+ CD56+ T cells; pDCs, plasmacytoid dendritic cells; MN, monocytes; MPH, macrophages. For all panels (**a-e**), bounds of box and whiskers represent the min-to-max cell count per 10^5 human CD45+ cells. Medians are indicated in each box as center line. For panel a and b, solid lines between box and whiskers link the mean of each box.

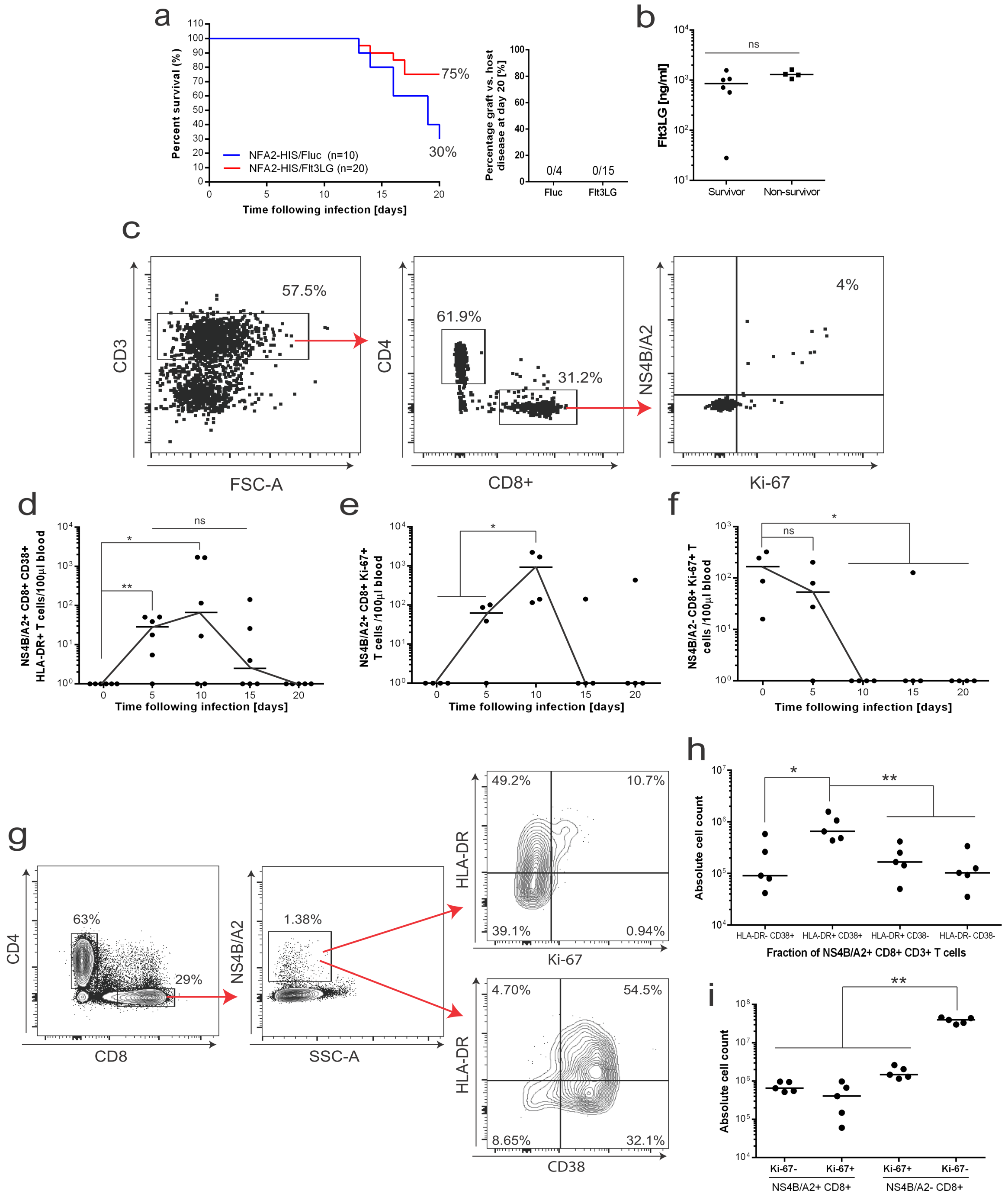

Supplementary Figure 8. Antigen-specific cellular responses upon YFV-17D infection in NFA2-HIS/Fluc and NFA2-HIS/Flt3LG mice.

a. Percent survival of NFA2-HIS/Fluc (blue, n=10) and NFA2-HIS/Flt3LG (red, n=20) mice over a course of 20 days after YFV-17D infection. The percentage of surviving NFA2-HIS/Fluc and NFA2-HIS/Flt3LG mice displaying signs of graft versus host disease (GVHD) at day 20 post infection are shown in the right panel. **b**. Human Flt3LG concentration in the serum of infected NFA2-HIS/Flt3LG mice that survived (survivor, closed circle) or not (non-survivor, closed square) the 20-day course of infection. Concentrations were measured 15 days following AdV-Flt3LG

injection. Medians are shown as horizontal black lines (n=10, 6 survivors and 4 non-survivors). Ns, non-significant (Wilcoxon-Mann-Whitney test). **c**. Representative flow cytometric plots displaying the gating strategy to identify and phenotype NS4B/A2+ CD8+ T cells in the peripheral blood of NFA2-HIS/Flt3LG mice over the course of YFV-17D infection. Relevant population frequencies are shown. **d-f.** Absolute cell count of NS4B/A2+ CD8+ CD38+ HLA-DR+ T cells (**d**), NS4B/A2+ CD8+ Ki-67+ T cells (**e**) and NS4B/A2- CD8+ Ki-67+ T cells (**f**) in the peripheral blood of NFA2-HIS/Flt3LG mice over the course of YFV-17D infection. Absolute counts are indicated as number of cells per 100 µl of blood. Medians are shown as horizontal black lines for each time point (n=4-5). *p ≤ 0.05, **p ≤ 0.01, ns, non-significant (Wilcoxon-Mann-Whitney test). **g**. Representative flow cytometric plots displaying the gating strategy to identify and phenotype NS4B/A2+ CD8+ T cells in the spleen of NFA2-HIS/Flt3LG mice at day 20 post YFV-17D infection. Relevant population frequencies are shown. **h.** Absolute cell count of NS4B/A2+ CD8+ T cells expressing different level of CD38 and HLA-DR in the spleen of NFA2-HIS/Flt3LG mice at day 20 post YFV-17D infection. Medians are shown as horizontal black lines (n=5). *p ≤ 0.05, **p ≤ 0.01 (Wilcoxon-Mann-Whitney test). **i.** Absolute cell count of NS4B/A2+ CD8+ T and NS4B/A2- CD8+ T cells expressing different level of Ki-67 in the spleen of NFA2-HIS/Flt3LG mice at day 20 post YFV-17D infection. Medians are shown as horizontal black lines (n=5). ** p≤ 0.01 (Wilcoxon-Mann-Whitney test).

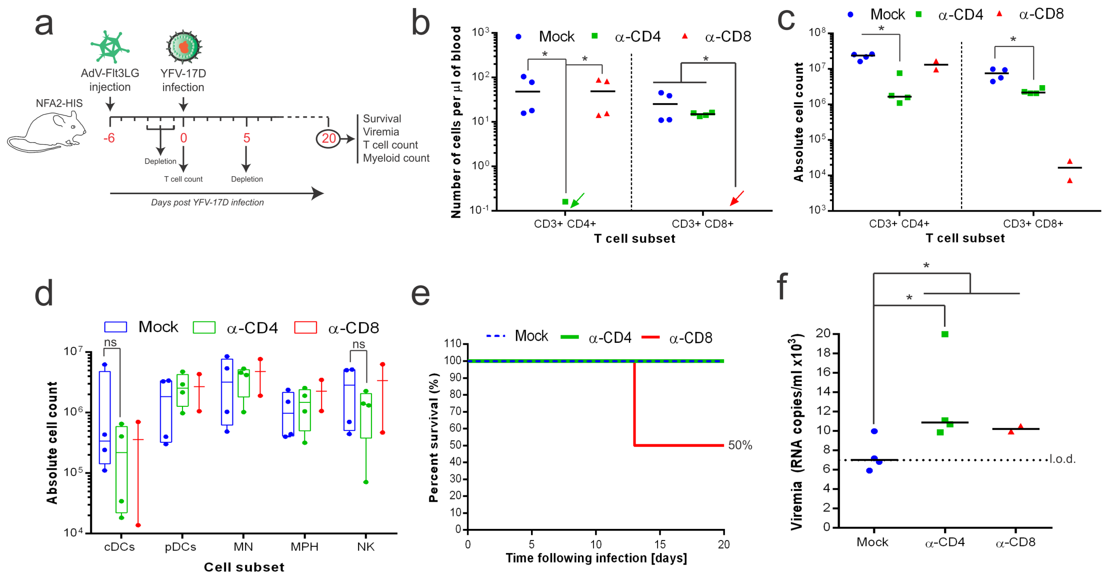

Supplementary Figure 9. T cell function in NFA2-HIS/Fluc and NFA2-HIS/Flt3LG mice during YFV-17D infection.

a. Schematic representation of the T-cell depletion experiment aiming at evaluating the contribution of CD4+ and CD8+ T cells in clearing YFV-17D infection in NFA2-HIS/Flt3LG mice. **b-c.** Absolute CD3+ CD4+ and CD3+ CD8+ T cell count in the peripheral blood (**b**) and spleen (**c**) of mock-treated (blue), α-CD4-treated (green) and α-CD8-treated (red) NFA2-HIS/Flt3LG mice prior YFV-17D infection (**b**) or at sacrifice (day 20 post infection, **c**). Absolute counts in the blood are reported as number of cells per 100 µl of blood. Medians are shown as horizontal black lines (n=4 per group). *$p \leq 0.05$ (Wilcoxon-Mann-Whitney test). **d.** Absolute cell count of human myeloid populations and NK cells in the spleen of mock-treated (blue), α-CD4-treated (green) and α-CD8-treated (red) NFA2-HIS/Flt3LG mice at time of sacrifice (day 20 post infection). Bounds of box and whiskers represent the min-to-max absolute cell count for each cell subset. Medians are indicated in each box as center line (n=4 per group). ns non-significant (Wilcoxon-Mann-Whitney test). cDCs, conventional dendritic cells; pDCs, plasmacytoid dendritic cells; MN, monocytes; MPH, macrophages; NK, natural killer cells. **e.** Survival of mock-treated (blue), α-CD4-treated (green) and α-CD8-treated (red) NFA2-HIS/Flt3LG mice over a course of 20-day of YFV-17D infection. Data are shown as percentage of survival. (n=4 per group). **f.** YFV-17D serum viremia in the peripheral blood of mock-treated (blue), α-CD4-treated (green) and α-CD8-treated (red) NFA2-HIS/Flt3LG mice at day 20 post YFV-17D infection. (+) RNA copies per ml were quantified by RT-qPCR. Limit of detection (dotted line) is shown. Horizontal black lines represent median viremia at each time point. (n=4 per group). *$p \leq 0.05$ (Wilcoxon-Mann-Whitney test).

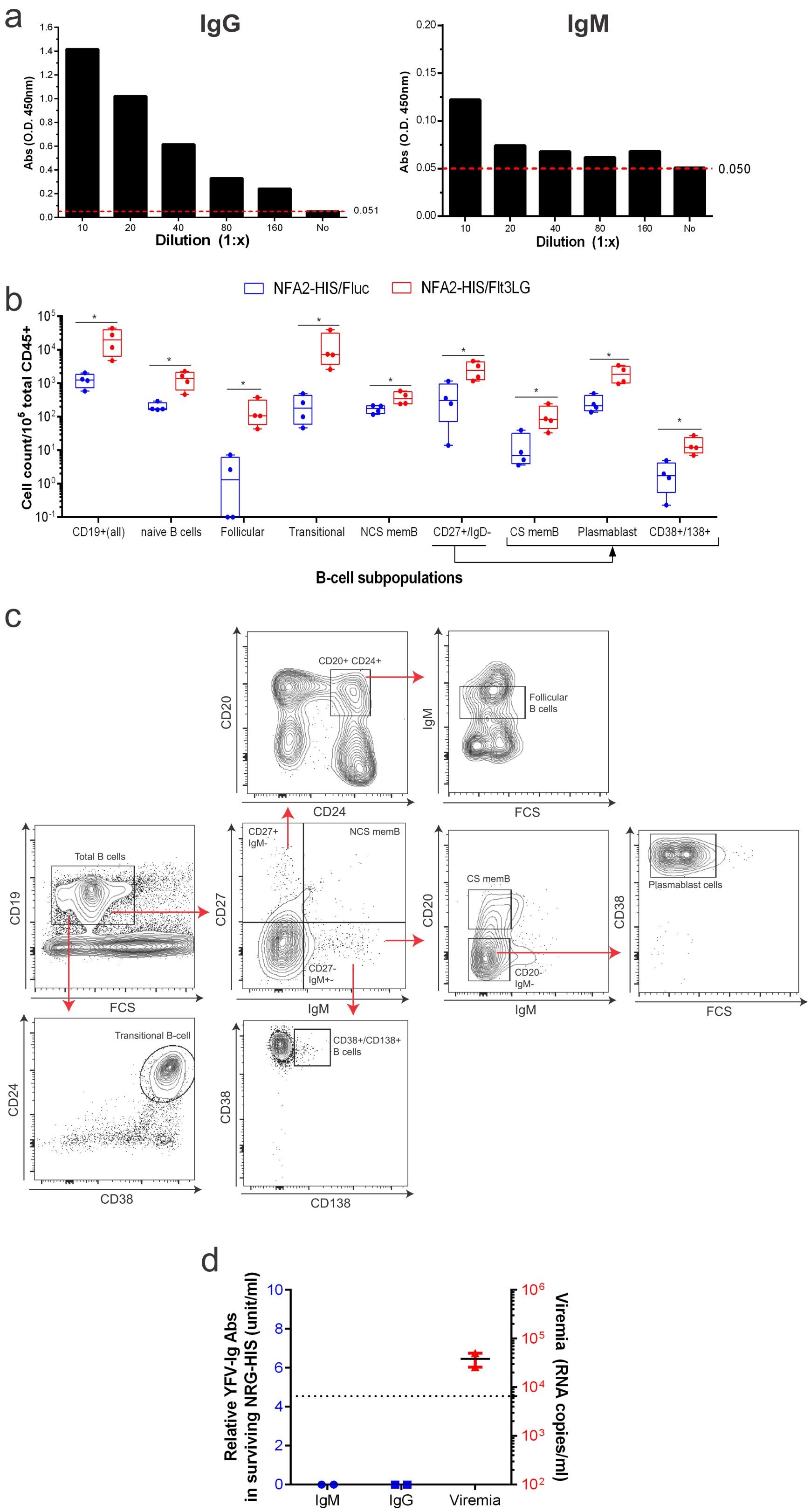
a
IgG
Abs (O.D. 450nm)
Dilution (1:x)
0.051
IgM
0.050
b
NFA2-HIS/Fluc
NFA2-HIS/Flt3LG
Cell count/10^6 total CD45+
CD19+(all)
naive B cells
Follicular
Transitional
NCS memB
CD27+/IgD-
CS memB
Plasmablast
CD38+/138+
B-cell subpopulations
c
CD20+ CD24+
Follicular B cells
Total B cells
CD27+ IgM-
NCS memB
CD27- IgM+-
CS memB
CD20- IgM-
Plasmablast cells
Transitional B-cell
CD38+/CD138+ B cells
d
Relative YFV-Ig Abs in surviving NRG-HIS (unit/ml)
Viremia (RNA copies/ml)
IgM
IgG
Viremia

Supplementary Figure 10. B-cell differentiation in NFA2-HIS/Fluc and NFA2-HIS/Flt3LG mice during YFV-17D infection. a. Relative quantification of human anti-YFV-17D IgG (**left**) or IgM (**right**) antibodies in the serum of a human vaccinee by ELISA. Several human serum concentrations were tested (from 1:10 to 1:160). A no Serum condition (No) was used as a negative control. Absorbance was measured at 450 nm. **b-c.** Quantification of multiple human B cell subpopulations in the bone marrow of NFA2-HIS/Fluc (blue) and NFA2-HIS/Flt3LG (red) mice at day 20 post infection. Frequencies of the different B cell subpopulations are shown in **b**. Bounds of box and whiskers represent the min-to-max cell count per 10^5 total CD45+ cells. Medians are indicated in each box as center line (n=4 per group). **p*≤0.05, ns non-significant (Wilcoxon-Mann-Whitney test). The gating strategy used to define these subpopulations is shown in **c.** Red arrows indicate the direction of the gating strategy: their tails are located within the parent population gate and their heads point to the parental population gated into. Follicular, follicular B cells; Transitional, transitional B cells; NCS memB, non-class switched memory B cells; CD27+/IgD-, CD27+ IgD- B cells; CS memB, class-switched memory B cells; CD38+/CD138+, CD38+ CD138+ B cells. CS memB, CD38+/CD138+ and plasmablast B cells are all CD27+ IgD-. **d.** Relative quantification of human anti-YFV-17D IgG or IgM antibodies (blue) in the serum of two surviving NRG-HIS mice (from a cohort of five mice) at day 40 post YFV-17D infection. Serum viremia (viral RNA copies per ml) for these two mice is indicated (red) and limit of detection is represented by a dotted line. Medians are shown as horizontal black lines (n=2).

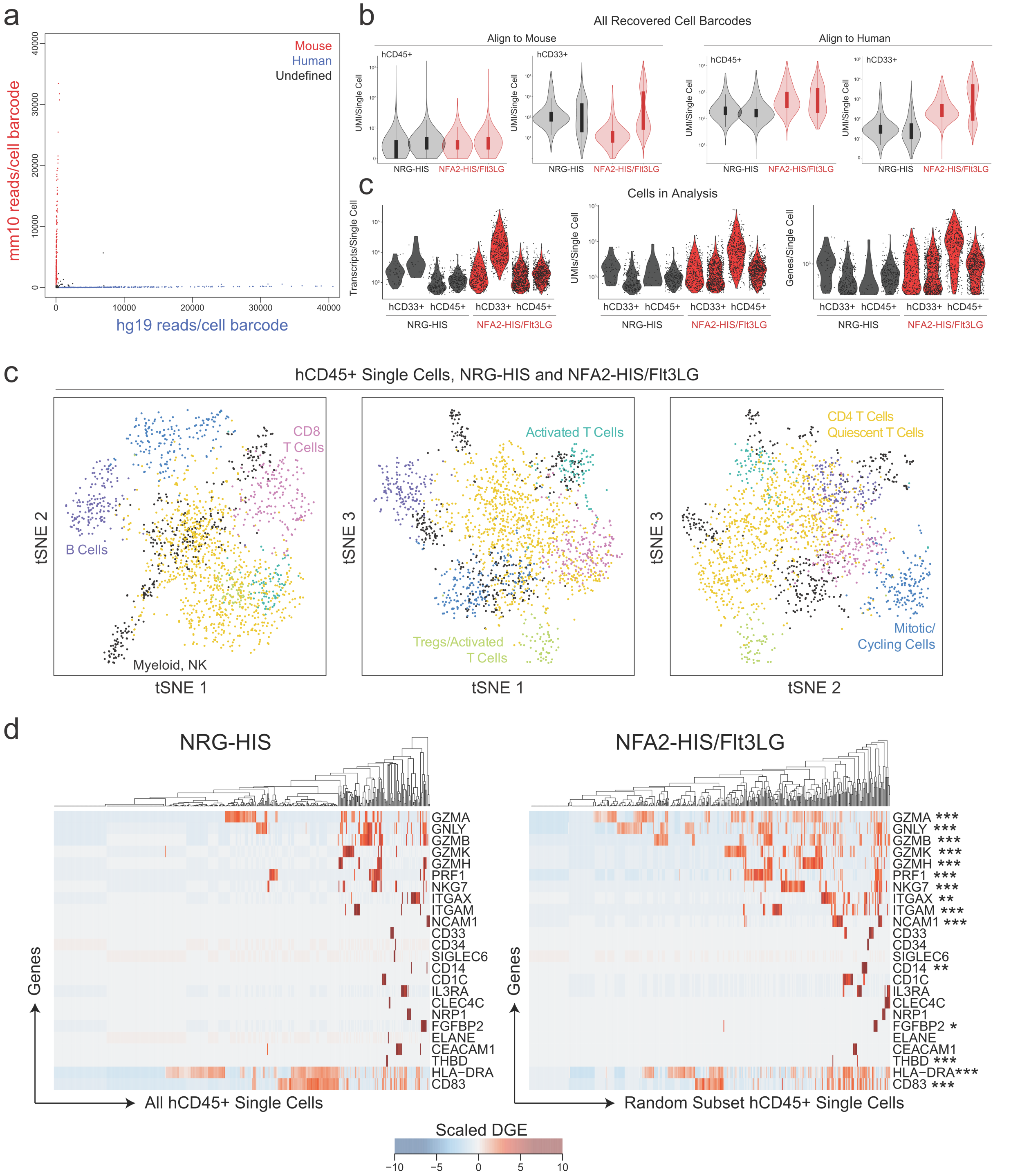

Supplementary Figure 11. scRNA-Seq profiling of NRG-HIS and NFA2-HIS/Flt3LG splenocytes: Data quality and cellular abundances.

a. Dual alignment to human and mouse genomes enables separation of humanized compartment from native mouse cells (representative plot from one NFA2 hCD45+ mouse). **b.** Transcript alignment to human (hg19) or mouse (mm10) transcriptomes by mouse and sort gate (hCD45+

or hCD33+) of all recovered cell barcodes. **c.** Hg19-aligned transcripts per cell (left), UMIs per cell (middle) and genes per cell (right) for all single cells retained for downstream analysis **d.** tSNE from all hCD45+ samples from both NFA2-HIS/Flt3LG mice and NRG-HIS mice analyzed together. Calculation of abundance of each cluster among either NFA2-HIS/Flt3LG or NRG-HIS mice is represented in **Fig. 9a,b**. **e.** Down-sampled datasets demonstrate significantly differentially expressed genes between NFA2-HIS/Flt3LG and NRG-HIS mice are not dependent on decreased scRNA-Seq cell count in NRG-HIS mice (Wilcoxon-Mann-Whitney test).

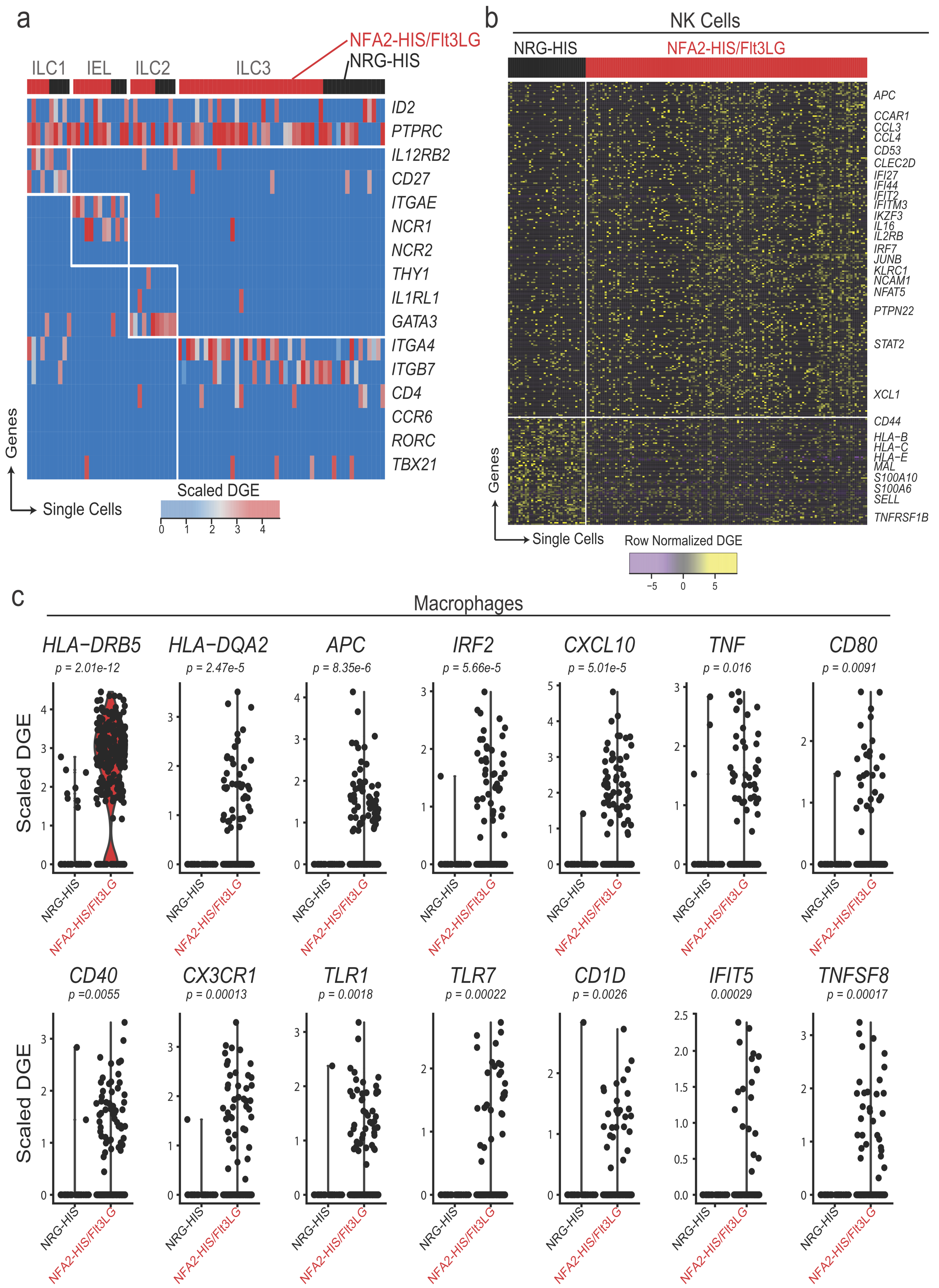
a
NFA2-HIS/Flt3LG
NRG-HIS
ILC1
IEL
ILC2
ILC3
ID2
PTPRC
IL12RB2
CD27
ITGAE
NCR1
NCR2
THY1
IL1RL1
GATA3
ITGA4
ITGB7
CD4
CCR6
RORC
TBX21
Genes
Single Cells
Scaled DGE
0 1 2 3 4
b
NK Cells
NRG-HIS
NFA2-HIS/Flt3LG
APC
CCAR1
CCL3
CCL4
CD53
CLEC2D
IFI27
IFI44
IFIT2
IFITM3
IKZF3
IL16
IL2RB
IRF7
JUNB
KLRC1
NCAM1
NFAT5
PTPN22
STAT2
XCL1
CD44
HLA−B
HLA−C
HLA−E
MAL
S100A10
S100A6
SELL
TNFRSF1B
Genes
Single Cells
Row Normalized DGE
−5 0 5
c
Macrophages
HLA−DRB5
p = 2.01e-12
HLA−DQA2
p = 2.47e-5
APC
p = 8.35e-6
IRF2
p = 5.66e-5
CXCL10
p = 5.01e-5
TNF
p = 0.016
CD80
p = 0.0091
CD40
p =0.0055
CX3CR1
p = 0.00013
TLR1
p = 0.0018
TLR7
p = 0.00022
CD1D
p = 0.0026
IFIT5
0.00029
TNFSF8
p = 0.00017
Scaled DGE
NRG-HIS
NFA2-HIS/Flt3LG

Supplementary Figure 12. Candidate ILCs and functional differences among splenic myeloid populations in NRG-HIS and NFA2-HIS/Flt3LG mice.

a. Heatmap showing candidate innate lymphoid cells (ILCs), separated by their expression of gene markers for ILC1, IEL, ILC2, and ILC3 subtypes. Red bars on the top of the heatmap identify cells from NFA2-HIS/Flt3LG splenocytes, black bars indicate cells from NRG-HIS splenocytes. **b.** Heatmap identifying differentially expressed genes between NFA2-HIS/Flt3LG-derived NK cells (red) and NRG-HIS-derived NK cells (black). All genes plotted represent a p-value ≤ 0.05. **c.** Violin plots of significantly differentially expressed genes between NFA2-HIS/Flt3LG-derived macrophages and NRG-HIS-derived macrophages.

SUPPLEMENTARY TABLES

		p_{adj} treshold		
Method	Model	**0.01**	**0.05**	**0.1**
Unbiased	NRG-HIS	0.0585	0.032	0.0091
	NRGF-HIS/Fluc	0.02975	0.04	0.0101
	NRGF-HIS /Flt3LG	0.11429	0.075	0.0317
Double selection	NRG-HIS	0.0967	0.043	0.077
	NRGF-HIS/Fluc	0.036	0.079	0.0232
	NRGF-HIS /Flt3LG	0.188	0.157	0.1392

Supplementary Table 1. Transcriptomic correlation index of humanized mouse models with human vaccinees. r correlation index for NRG-HIS, NRGF-HIS/Fluc and NRGF-HIS/Flt3LG are reported for three distinct p_{adj} threshold (0.01, 0.05 and 0.1) and for a given comparative method (unbiased and adjusted).

SUPPLEMENTARY NOTES

Supplementary Note 1. Analysis of the diversity and functionality of the immune system in humanized mice by single-cell RNA sequencing analysis.

We used single-cell RNA-Seq (scRNA-Seq) to analyze in greater depth the complexity and cellular diversity of the HIS in YFV-17D vaccinated conventional (NRG-HIS) and refined humanized mice (NFA2-HIS/Flt3LG). scRNA-Seq allows unbiased molecular profiling of individual immune cells to identify cell states and their associated signatures and enables precise quantitation and analysis of rare cell types from complex samples. Here, we employed Seq-Well [52]—a recently developed platform for massively parallel scRNA-Seq that utilizes a microwell-based system to perform mRNA capture and library generation from a heterogeneous collection of cells—to capture the complexity and diversity of the human immune system of NFA2-HIS/Flt3LG mice and conventional NRG-HIS mice, which only mount a poor cellular and transcriptomic response to YFV-17D.

We isolated splenocytes from two of the NFA2-HIS/Flt3LG mice that mounted a specific humoral response to YFV-17D (**Fig. 7e-f**) at six-weeks post YFV-17D infection, and sorted these cells by human CD45 (hCD45) or human CD33 (hCD33) expression. For a direct comparison with conventional humanized mouse model, we infected a cohort of five NRG-HIS mice. Among those, two mice survived the six weeks of infection (survival rate of 40%), but survival did not correlate with viral clearance or with the presence of YFV-specific antibodies (**Supplementary Figure 10d**), suggesting that the mouse myeloid compartment could play a role in a partial control of YFV-17D infection in NRG-HIS mice. Such hypothesis is supported by the strong difference in survival between NRG-HIS mice and NFA2-HIS/Fluc over a 20-days course of infection (90% vs. 30%) (**Supplementary Figure 1a; Supplementary Figure 8a**). Hence, we isolated splenocytes from the two-surviving NRG-HIS mice six weeks post infection, and sorted these cells similarly to NFA2-HIS/Flt3LG splenocytes.

We ran parallel Seq-Well arrays for each mouse and sort, thus enabling both unbiased characterization of the relative abundances of all lymphocytes, as well as a deeper examination of the cellular diversity within the myeloid compartment. We aligned the single-cell transcriptomes to both mouse and human reference annotations to differentiate engrafted from endogenous immune systems (**Supplementary Figure 11a**). First, we observed that there was a difference in the quantity of viable human single cells that passed initial quality filtering between NRG-HIS and NFA2-HIS mice (**Supplementary Figure 11b**). To confirm that the difference between experimental groups was not due to discrepancies in sequencing depth or another technical factor, we compared alignment statistics among the mouse single-cell transcriptomes that persisted in both experimental groups as an internal control. We found that the abundance and quality of "contaminating" mouse single cells were equivalent between both NRG-HIS and NFA2-HIS/Flt3LG mice, while the abundance of high-quality human single cells was substantially lower in NRG-HIS mice.

On the cells that passed quality thresholds, we performed dimensionality reduction and graph-based clustering to distinguish cellular subpopulations, represented as clusters in tSNE plots. We identified cluster-defining gene sets and surveyed these for lineage and cell type-defining genes to annotate each subpopulation (**Supplementary Data 2**). First, we examined the cell types identified in hCD45+ single cells in NFA2-HIS (**Fig. 8a**) compared to NRG-HIS (**Fig. 8b**). Both groups contained high abundances of quiescent/naïve CD4 T cells, yet NFA2-HIS splenic CD45+ cells showed a higher diversity of well-resolved subpopulations of activated and differentiated T lymphocytes, including higher frequencies of cytotoxic lymphocytes (by expression of *CD8A*, *CD8B* and numerous granzymes and perforin), T cells expressing known activation and memory markers (*CD27, CCR7, STAT1, CD40LG*), and regulatory T cells (distinguished by high expression of *FOXP3*, *CTLA4*). B cells were found to comprise a higher percentage of the

hCD45+ pool in NRG-HIS compared to NFA2-HIS mice. However, such differential frequency inversely correlated with the B-cell response we measured (**Fig. 7e,f; Supplementary Figure 10d**) as NFA2-HIS/Flt3LG developed a neutralizing antibody response to YFV-17D. Critically, the abundance of myeloid and NK cells within the hCD45+ samples was significantly higher in NFA2-HIS/Flt3LG mice, and we were unable to resolve a distinct cluster of myeloid cells from the NRG-HIS populations when clustered alone.

To more directly compare the myeloid compartment between the NRG-HIS mice and the NFA2-HIS mice, we combined both hCD33+ and hCD45+ samples from either the NFA2-HIS mice (**Fig. 8c**) or NRG-HIS mice (**Fig. 8d**), and computationally "gated out" all T cells and B cells by expression of TCR or BCR related genes (the full list can be found in the **Methods** section). Again, we observed dramatic differences in the recovery of high quality human single cell transcriptomes between both mouse models. In NFA2-HIS/Flt3LG mice, we identified 6 distinct clusters of cell types corresponding to subpopulations of NK cells, monocytes, macrophages, cDCs, pDCs, and cross-Presenting DCs (full cluster-defining genes in **Supplementary Data 2**). The three DC subpopulations were annotated primarily by their expression of *CD1C* (as cDCs), *IL3RA* and *CLEC4C* (pDCs), and *XCR1* and *CLEC9A* (cross-Presenting DCs). Monocytes and Macrophages were differentiated principally by the acquisition of functional characteristics such as *C5AR1*, *HLA-DRA*, *LYZ,* and *IL8* expression. NK cells, meanwhile, displayed broad expression of functional markers such as *IL2RB, GNLY, GZMA, KLRB1, CCL5,* as well as *NKG7 and NCAM1*.

We directly compared the expression of the top subpopulation-defining genes in NFA2-HIS non-T, non-B single cells (**Fig. 8e**) with the corresponding single cell gate in NRG-HIS mice (**Fig. 8f**), revealing significant differences in expression between similar immune population from each mouse models (in all cases, significance indicated increased expression of a given marker among NFA2-HIS mice). Especially, several NK and cDCs activation markers were significantly upregulated in NFA2-HIS/Flt3LG mice in comparison to NRG-HIS (such as *HLA-DRA*, *CD83* and *CD40* for cDCs, and *CCL5, GNLY, GZMA, PRF1* and *CD226* for NK cells) (**Fig. 8e,f**), hence strengthening evidence of a superior innate immune response in NFA2-HIS/Flt3LG mice. To confirm skewed abundances in immune subtypes between NRG-HIS mice splenocytes and splenocytes from NFA-HIS/Flt3LG mice, we completed all above methods for dimensionality reduction and clustering on a merged dataset of all hCD45+ single cells from both NRG-HIS and NFA2-HIS mice **(Supplementary Figure 11c**). By analyzing within a single PCA and tSNE space, we were then able to directly compare cell type abundances between experimental groups (**Fig. 9a, b**). When analyzed alone, hCD45+ NRG-HIS single cells did not yield a distinct subpopulation that could be annotated as a myeloid type cluster. However, when these cells were clustered in concert with abundant myeloid cell types from the NFA2-HIS mice, we could resolve these cells distinctly (previously, they were mis-clustered with other cell subpopulations). Each cell type represented in **Fig. 9a, b** was annotated as above—by identifying significant cluster-defining gene sets and comparing these to known cell markers and literature queries—with the exception of NKT cells. We distinguished NKT cells, which naturally clustered with either NK cells, CTLs, or Activated T Cells, by identifying single cells that express CD3 genes and *NCAM1*, display absent expression of non-NKT TCR genes (non-*TRAV24* or -*TRBV11*), *CD8A, and CD8B*.

Next, we tested differential expression between hCD45+ single cells in both mouse models over a curated list of lineage and cell-type relevant genes (**Fig. 9c, d**). This revealed significantly higher expression of functional markers of NK and activated T cells, notably via elevated expression of *NKG7* and *NCAM1*, confirming better reconstitution of NK cells in NFA2-HIS/Flt3LG mice. We also observed higher expression of *ITGAM, CD14, CD83, FGFBP2, THBD,* and *HLA-DRA* – all suggesting higher abundance of functional, mature, myeloid cells in NFA2-HIS/Flt3LG mice. We did not, however, recover high numbers of granulocytes, possibly due to the freeze-thaw cycle of the samples. We confirmed these differences were not simply explained by higher overall abundance of cells within the NFA2-HIS mice by randomly subsampling the NFA2-HIS

dataset to match the number of single cells in the NRG-HIS dataset and recalculating differential expression metrics (**Supplementary Figure 11d**).

While definitive RNA markers for innate lymphoid cells (ILCs) have not yet been defined, we attempted to identify different ILC subsets (ILC1, ILC2, ILC3 and IEL, i.e. intraepithelial lymphocytes) in our scRNA-Seq data. We first excluded all cells with expression of known lineage-defining markers for alternative cell types. The full gate employed is *TCR-CD3D-CD3E-CD3G-CD34-KIT-NCR2-BCR-MS4A1-CD79A-CD79B-CD19-ITGAX-CD14-CD33-ELANE-CLC-IL3RA-CLEC4C-ITGAM-CD1C-NCAM1-NKG7-KLRB1-.* This yielded 148 cells, represented in each of the 4 mice; all within the CD45+ sorted conditions. We next clustered our single cells according to their expression of known markers to define ILC1, ILC2, ILC3, and IELs. We putatively named our single cells as ILC1 if they showed high co-expression of *IL12RB2* and *CD27*, IELs if they expressed *ITGAE*, *NCR1*, or *NCR2*, ILC2 if they expressed *THY1*, *IL1RL1* and *GATA3*, and ILC3 if they expressed *ITGA4*, *ITGB7*, *CD4*, *CCR6*, *RORC*, and/or *TBX21*. We have plotted this data as a heatmap, showing single cell expression of each of these markers, as well as *ID2* and *PTPRC* (CD45) (**Supplementary Figure 12a)**. Using this method, we putatively identified 10 ILC1 cells, 13 IELs, 11 ILC2s, and 47 ILC3s. We did not identify significant differences in the abundance of any ILC subtype between NRG-HIS and NFA2-HIS/Flt3LG animals. However, we noted major caveats and pitfalls in this approach for ILC gating in single cell RNA-Seq data. First, we are relying on the absence of specific mRNA species captured to identify cells as potential ILCs. ScRNA-Seq data, especially when collected using high-throughput methods, is subject to high rates of gene drop-out, and we cannot exclude the possibility that these cells actually represent other cell types. We anticipate the ILC numbers we identify are likely an overinflated count of the true abundance within the dataset. Second, for partitioning candidate ILCs into ILC1, ILC2, ILC3, and IEL subsets, we rely on marker genes used for Flow Cytometry, but not confirmed as definitive for scRNA-Seq data. Third, since mRNA abundances may not match protein abundances on the cell surface (due, for example, to differences in the timing of transcription and translation, the stability of mRNA and proteins or protein complexes), we are further subject to both false positives and negatives. We did not identify significant differences in the abundance of any inferred ILC subtype between NRG-HIS and NFA2-HIS/Flt3LG animals.

Finally, we isolated NK cells and macrophages from NFA2-HIS/Flt3LG and NRG-HIS scRNA-Seq libraries to complete an unbiased, whole-transcriptome differential expression analysis between matched cell types to assess in more detail the phenotypic and potentially functional alterations that arise in each model (**Supplementary Figure 12b,c**). Unfortunately, there are not sufficient DCs in the NRG-HIS mice to complete a statistically rigorous differential expression analysis. We utilized the R software package SCDE (https://bioconductor.org/packages/release/bioc/html/scde.html) for assessment of differential expression, and full results can be found in **Supplementary Data 3.** Among the NK cells, we identified 99 genes significantly upregulated ($p \leq 0.01$) in NFA2-HIS/Flt3LG mice, and 20 genes upregulated in NRG-HIS mice. Splenic NK cells in NFA2-HIS/Flt3LG mice expressed higher abundances of chemokines *CCL3*, *CCL4*, and *XCL1*, consistent with enhanced activation. NK cells from NFA2-HIS/Flt3LG mice also showed upregulation of antiviral/interferon-induced genes such as *IFI27*, *IFI44*, *IFIT2*, *IFITM3*, and *IRF7*. Interestingly, NRG-HIS splenic NK cells expressed higher abundances of Class I HLA molecules, L-selectin (*SELL*), and *CD44*, implicating unique activation and homing phenotypes among NK cells in this model. Differential expression analysis yielded 18 genes upregulated in NRG-HIS macrophages, and 1434 genes upregulated in NFA2-HIS/Flt3LG Macrophages ($p \leq 0.01$). Similar to NK cells, NFA2-HIS/Flt3LG-dervied Macrophages expressed higher levels of functional and activation markers such as *TNF*, *IRF2*, *CXCL10*, *CD80*, *TLR7*, *TLR1*, *CD1D*, and *CD40*. NRG-HIS-derived macrophages were generally quiescent and express very few markers associated with activation or functional purposes.

Altogether, we demonstrate here that scRNA-Seq can provide a unique and highly-resolved picture of the human immune system reconstitution and diversity in humanized mice. These data

also confirm the robust engraftment and functionality of the myeloid and NK/NKT cell compartment in NFA2-HIS/Flt3LG mice, and consequently the importance of these cellular compartments for an enhanced systemic human immune response against a potent human vaccine.
